# Development and validation of risk profiles of West African rural communities facing multiple natural hazards

**DOI:** 10.1371/journal.pone.0171921

**Published:** 2017-03-01

**Authors:** Daniel Asare-Kyei, Fabrice G. Renaud, Julia Kloos, Yvonne Walz, Jakob Rhyner

**Affiliations:** United Nations University, (UNU-EHS), UN Campus, Platz der Vereinten Nationen 1, Bonn, Germany; INDEPTH Network, GHANA

## Abstract

West Africa has been described as a hotspot of climate change. The reliance on rain-fed agriculture by over 65% of the population means that vulnerability to climatic hazards such as droughts, rainstorms and floods will continue. Yet, the vulnerability and risk levels faced by different rural social-ecological systems (SES) affected by multiple hazards are poorly understood. To fill this gap, this study quantifies risk and vulnerability of rural communities to drought and floods. Risk is assessed using an indicator-based approach. A stepwise methodology is followed that combines participatory approaches with statistical, remote sensing and Geographic Information System techniques to develop community level vulnerability indices in three watersheds (Dano, Burkina Faso; Dassari, Benin; Vea, Ghana). The results show varying levels of risk profiles across the three watersheds. Statistically significant high levels of mean risk in the Dano area of Burkina Faso are found whilst communities in the Dassari area of Benin show low mean risk. The high risk in the Dano area results from, among other factors, underlying high exposure to droughts and rainstorms, longer dry season duration, low caloric intake per capita, and poor local institutions. The study introduces the concept of community impact score (CIS) to validate the indicator-based risk and vulnerability modelling. The CIS measures the cumulative impact of the occurrence of multiple hazards over five years. 65.3% of the variance in observed impact of hazards/CIS was explained by the risk models and communities with high simulated disaster risk generally follow areas with high observed disaster impacts. Results from this study will help disaster managers to better understand disaster risk and develop appropriate, inclusive and well integrated mitigation and adaptation plans at the local level. It fulfills the increasing need to balance global/regional assessments with community level assessments where major decisions against risk are actually taken and implemented.

## 1. Introduction

Africa is currently a continent under pressure from multiple stresses and is highly vulnerable to the impacts of climate change [1,2]. Fields [3] argues that the influence of multiple stressors such as environmental disasters, infectious disease, economic turbulence from globalization, resource privatization, and civil conflicts, combined with the lack of resources for adaptation, will present serious challenges for African communities struggling to adapt to climate change. West Africa in particular, has been described as a hotspot of climate change [2]. In this region a temperature of 3–6°C above the late 20th century baseline is “*very likely”* to materialize within the 21st century and the fact that this projection is expected to occur one or two decades earlier than other regions [2] contributes to making the region even more vulnerable to climate change. The frequency of occurrence of extreme events is expected to increase and the interaction of climate change with non-climate stressors will aggravate vulnerability of agricultural systems in semi-arid Africa such as the West Sudanian Savanna region of Burkina Faso, Ghana and Benin [2]. There is also medium confidence that projected increase in extreme rainfall will “contribute to increases in rain-generated local flooding” ([4], p. 24). For West Africa, Sylla *et al*. [5] projected a decrease in the absolute number, but an increase in the intensity of very wet events–leading to increased drought and flood risks towards the late 21st century. Increases in the frequency and intensity of extreme weather events constitute an immediate and damaging impact of climate change [6].

Yet, comprehensive and quantitative understanding of the vulnerability and risk faced by West African rural communities to these multiple hazards, including the commonly occurring hazards of floods and droughts are still lacking. The few studies available in the area have either qualitatively assessed vulnerabilities (e.g. [7, 8]) or only looked at specific aspects such as vulnerability to food insecurity [9,10], or focused on single hazards such as floods (e.g. [11,12]). Asare-Kyei *et al*. [13] reviewed vulnerability and risk indices developed at different scales from local to national assessments (see for example [14, 15, 16, 17,18,19,20]). All these studies have measured vulnerability, resilience and adaptation using a variety of concepts, approaches, and indicators, however, important considerations such as applicability to local communities, methods to estimate localized risks, inclusion of at risk populations in developing the indicators themselves, use of multiple hazards and multiple scales were often missing [13,21]. Studies such as Linstädter *et al*. [22] assess the resilience of pastoral SES to droughts in South Africa whilst Martin *et al*. [23] assessed livelihood loss to drought using a model based approach. Although these recent studies introduce new and interesting dimensions to resilience assessment in the context of droughts; using multidisciplinary approaches [22] and scenario comparison [23], they do not integrate multiple hazards occurrence, and limit their assessment to pastoral systems. For West Africa, Asare-Kyei *et al*. [13] found that, “no study has attempted to understand the risk patterns of rural communities in the context of climate change” through a set of participatory developed indicators. The only study that comes close is provided by the United States Agency for International Development [17], however, indicators were derived purely from literature without a participatory process with the vulnerable themselves. For more information of available risk and vulnerability indices, see Asare-Kyei *et al*. [13,21].

Studies such as Welle *et al*. [24] and Beckmann *et al*. [25] have also developed risk indices across countries and compared countries with high and low risk levels. However, it has been found that studies that use the same indicator set and make an effort to derive relative vulnerabilities across countries produce results that may be contradictory to expert knowledge [26]. The World Development Report in 2010 reviewed two major vulnerability-driven indices–Disaster Risk Index, DRI [20] and Index of Social Vulnerability to Climate Change for Africa, SVA [27] and concluded that these indices created spatial patterns out of tune with development-driven indicators and consistently showed a pattern contradictory to expert knowledge [26]. This was corroborated by Asare-Kyei *et al*. [13] that such contradictory results are expected because using the same indicators ignore the salient indicators deemed to be relevant by the local populations. In countries where the same indicators apply, they differ in their ranking and hence the weights that must be applied in estimating the final risk index. To this end, this study does not intend to use common indicators and make comparisons across countries but rather uses a participatory bottom-up approach where case study specific indicators are used.

In 2007, Birkmann [28] indicated that a discussion has just begun as to whether and how global approaches and the associated indicators can be down-scaled to estimate localized risk and vulnerability and whether they provide appropriate and useful information. However, to date, little is known about the risk profiles of rural West African communities particularly regarding risk to multiple hazards. Yet, it is acknowledged that risk and vulnerability identification and measurement before and after the occurrence of hazards are essential tasks for effective and long term Disaster Risk Reduction (DRR) [28]. There is an increasing need to balance global, regional and sub-national assessments with community level assessments because these are the scales where major decisions against disaster risk reduction are made and expected to be implemented. A common methodology to identify and measure risk and vulnerability to climatic hazards in order to define disaster risk reduction measures is still not sufficiently developed [28,29]. To this end, participatory “bottom–up” methods are increasingly being employed to identify and document the processes that occur at a local level, involving decision-makers in communities and societies [13,30,31,32].

However, despite the growing acknowledgment of the necessity of community participation for sustainable disaster reduction, this has not been translated into actions to carry out participatory community based vulnerability and risk assessments in the West African sub region. In this study, a community based participatory method of assessing risk to multiple natural hazards based on indicators is introduced to address the gaps enumerated above.

Validation or model evaluation is an essential aspect of assessing the accuracy of complex model outcomes. Gall [33] outlined six critical dimensions of model evaluation, of which validation is a key component. However, in almost all risk assessment studies reviewed, the only validation approach is based on statistical assessments of model intrinsic uncertainties. Damm [14] observed that the development of indicators and subsequent modelling of composite risk indices have inherent uncertainties due to the many subjective decisions made by authors, yet “conventional validation of vulnerability is not possible as vulnerability cannot be measured in the traditional sense” and concluded that “validation still remains an open challenge” in risk assessment (Damm [14], p.17, 197). To this end, major risk assessments studies such as the World Risk Index [24,25,34,35] used statistical Monte Carlo analysis and sensitivity analysis as validation tools. Other studies such as Adger & Vincent [36] and Brooks *et al*. [37] attempted to undertake indicator validation using mortality outcome. On the other hand, the difficulties with validating complex risk assessment models means that some studies don’t undertake any validation at all, e.g. [29]. To address this open challenge in risk assessment, the study introduces the concept of community impact score (CIS) to validate the indicator-based risk and vulnerability modelling. The CIS is a novel and innovative approach to validate risk assessment and uses observed disaster impacts to validate the results of a complex indicator aggregation model. The result of this aggregation model is termed in this study as the West Sudanian Community Risk Index (WESCRI). The contributions of single constituent parameters to WESCRI describe the specific risk profile of a community in terms of the main determinants of risk.

This study aims at (1) conducting risk assessment for multiple hazards (drought and floods) through a bottom-up participatory process as opposed to the classical top-down, large scale approaches; (2) assessing risk from the perspectives of a coupled SES rather than single-hazard-decoupled risk assessments; (3) quantifying risk using indicators relevant for rural communities to understand the constituents (profiles) of risk across community clusters within a watershed and (4) exploring an innovative validation approach for risk assessment.

This disaster index across community clusters helps to identify and support decision-makers with information to recognize and map risk hotspots even within communities in a single watershed in order to support priority setting for risk-reduction strategies. Three case studies are presented for three watersheds in three different countries in West Africa. The study helps to provide a better understanding of the risks and vulnerabilities of these rural communities and helps to differentiate between communities by the elements characterizing their risks and vulnerabilities. Studying risk profiles of rural communities also provides an insight on how to situate vulnerability, risk and climate change adaptation efforts within the context of the community’s sustainable development agenda and can help to develop appropriate, inclusive and well integrated mitigation and adaptation plans at the local level.

## 2. Research sites

**Fig 1:**
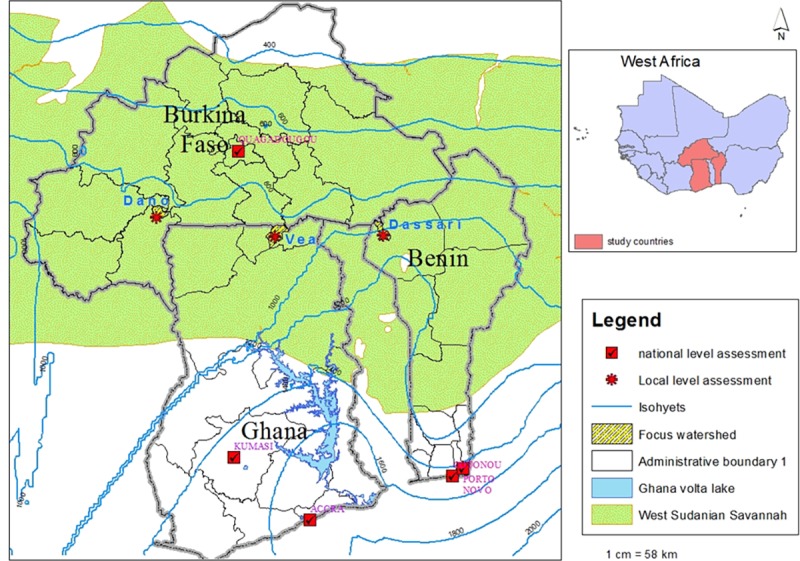
Overview of the West African study sites. Showing also the three watersheds which are presented in detail in S1 File.

Within the structure of the West African Science Service Centre for Climate Change and Adapted Land Use (WASCAL) project, three study areas in three West African countries have been selected. These areas are (i) the Vea area in the Upper East region of Ghana; (ii) the Dano area in the province of Sud-Ouest of Burkina Faso; and (iii) the Dassari area in the commune of Materi in north-west Benin (Fig 1). These study areas, which belong to the Sudanian Savanna ecological zone, have similar climate and are under varying forms of agricultural systems. The areas are predominantly rural and have relatively high population density compared to other regions in the countries [38].

The study areas were delineated into community clusters based on high resolution land use maps developed by Forkuor *et al*. [39]. The community clusters were used as the unit of analysis for the spatially explicit vulnerability and risk assessment. The delineation into community clusters which is explained in detail in Asare-Kyei *et al*. [38] was based on a digital elevation model (DEM), river channel systems, populations in the communities or population conglomerations, community groupings by local authorities, settlement structures as well as the operational plans which are used by local disaster managers to segregate and demarcate the areas for effective disaster management

In the Vea study area, 13 community clusters were delineated. The largest of these clusters is the Kula River drain (Fig A in S1 File), named after the Kula river which is well known for causing many of the floods in the area. Other major community clusters are the Vea main drain and Kolgo/Anateem valley. These community clusters are located at the downstream of the Vea and Kolgo Rivers and are also significantly exposed to floods. Similarly, the Dano study area has further been delimited into 13 community clusters. The Yo, Bolembar, Gnikpiere and Loffing-Yabogane are the major clusters with extensive river system, smallholder agriculture and many scattered settlements and hamlets. The Dassari area in Benin was also delineated into 12 community clusters. The Sétchindiga, Porga and Nagassega community clusters are most prominent as they are crossed by a major river network that significantly exposes the area to flooding. Details about the procedure for the community clustering can be found in Asare-Kyei *et al*. [38]. In Table 1, the physical characteristics of the three watersheds are presented. Other information about flood and drought events in the watersheds are presented in the supplementary information, S1 File.

**Table 1:** Physical characteristics of the three watersheds.

Watershed	Average annual rainfall (mm/year)	Average peak runoff (M^3^/sec)	Evapotranspiration (mm/year)	Mean slope (%)
**Vea**	980	155.70	1455	0.4
**Dano**	910	68.96	1747	0.5
**Dassari**	1000	113.11	1552	0.3

Data source: runoff data from Asare-Kyei *et al*. [38], other data from Ibrahim *et al*. [40].

Field observations and interactions with people in the communities revealed that all these communities are frequently exposed to droughts and floods and life in these communities has been reduced to routine coping or adaptation to these two hazards. The sustainability of a household’s livelihood now depends on the household’s ability to manage the impacts of drought and flood events. S1 File in the supporting information section give details about each of the study areas.

## 3. Methods

**Fig 2:**
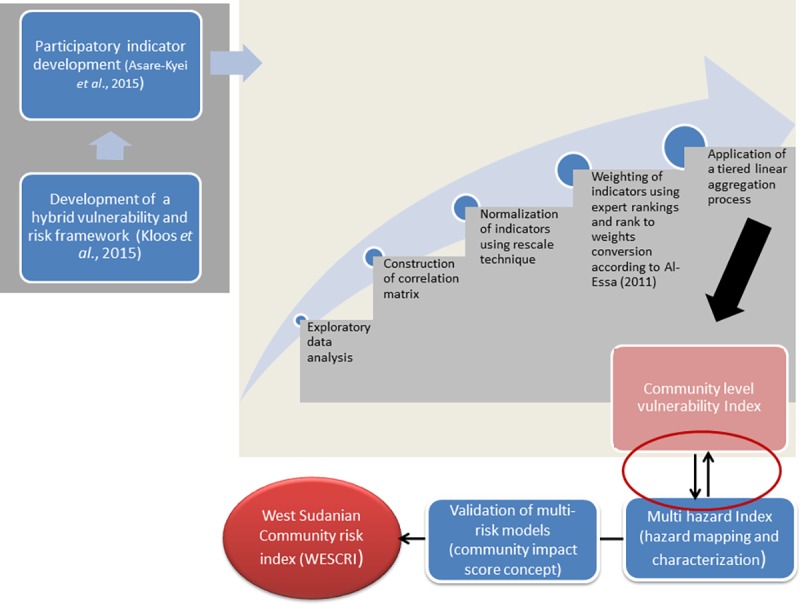
A stepwise process to quantify risk and vulnerability at the community level.

A stepwise process (Fig 2) was followed, first to develop the community level vulnerability index and subsequently the West Sudanian Community Risk Index (WESCRI). The sections below present detailed descriptions of these work steps.

### 3.1. Development of a multi-hazard vulnerability and risk assessment framework

In this study, an attempt was made to conduct the first operationalization of the framework proposed by Kloos *et al*. [41] at the community level in three West African countries. The framework is based on the key element, a SES, reflecting the connections and feedbacks between the environmental and social sub-systems taking place at various spatial scales (local, sub-national and national) [41]. Multiple temporal scales of different components of the framework are also covered by looking at the dynamics within the system.

**Fig 3:**
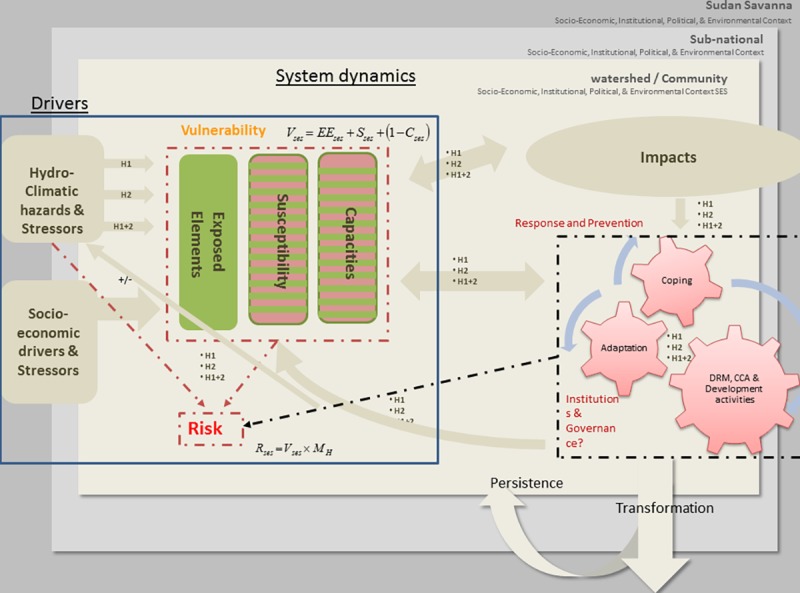
The Proposed West Sudanian Savannah Vulnerability framework by Kloos *et al*. [41].

Risk is to be evaluated against hydro-climatic hazards and stressors (Fig 3), which may materialize as sudden shocks such as floods and/or heavy rainfall events, slow onset events such as droughts, late onset of the rainy season but also more gradual changes such as changes in variability or averages of rainfall. At the same time, an SES is affected by socio-economic drivers and stressors (Fig 3) which may lead to environmental changes that can turn into stressors or hazards in themselves.

Ecosystem services are integral to the SES and provide numerous monetary and non-monetary benefits to people living in the system [42]. To account for the multi-hazard nature, two hazards are introduced to the framework, ‘H1’ and ‘H2’, and the combination of both hazards selected for the West Sudanian Savanna case, ‘H1+H2’ representing floods and droughts. For further details on the framework, see Kloos *et al*. [41].

In this framework, vulnerability is characterized by exposure, susceptibility and the capacity of the coupled SES to cope and adapt to the impacts of either a single hazard or the combined effects of multiple hazards. Risk is a product of vulnerability and the characteristics of the hazard. Characteristics of the hazards in this study are construed to mean the intensity and frequency of occurrence of the two hazards, floods and droughts.

Studies such as Beck *et al*. [34] and Welle *et al*. [24] have included the exposure term in risk quantification and there have been debates as to whether exposure should be included in vulnerability component or the risk term [15]. In this study however, the point of departure from the framework proposed by Kloos *et al*. [41] is that exposure is only construed to mean the elements of the SES that are exposed to the multiple hazards, hence the term ‘Exposure’ as used by Kloos *et al*. [41] is replaced with ‘Exposed Elements’. This conceptualization helps to provide an avenue to deal with the debate on whether exposure should be part of vulnerability or included in the risk term. According to Birkmann ([15], p.38), “an element or system is only at risk if the element or system is exposed and vulnerable to the potential phenomenon”. Although exposure is often related to the hazard, excluding exposure from vulnerability assessment entirely makes such an analysis “politically irrelevant” ([15], p.38). This is because once vulnerability is agreed to mean those conditions that intensify the susceptibility and decrease the capacity of the SES to the impact of the hazard, it also rests on the spatial dimension, by which the degree of exposure of the SES to the hazard is referred to [15,16]. This study is based on the assertion of Birkmann [15], that the location’s general exposure is essentially a component of the hazard whilst the degree of exposure of its critical elements such as farmlands, schools, houses etc. falling in hazard prone areas indicates the spatial dimension of vulnerability. In this study therefore, this spatial dimension of vulnerability is termed as ‘Exposed Elements’ and shows that exposure is a partial characteristic of vulnerability. To this end, indicators used to describe the SES spatial dimension of vulnerability in this study include: agricultural areas in hazard zones, insecure settlements (share of the area’s settlement intersecting the hazard zones), protected areas in hazard zones, agricultural dependent population, etc.

From these conceptualizations, vulnerability (*V*) and risk (*R*) of the SES can be expressed as:

$$V_{\mathrm{ses}}={\mathrm{EE}}_{\mathrm{ses}}+S_{\mathrm{ses}}+\left(1-C_{\mathrm{ses}}\right)$$ (1)

$$R_{\mathrm{ses}}=V_{\mathrm{ses}}\times M_{H}$$ (2)

where *V* is the vulnerability of the SES, *EE* is the exposed elements within the SES indicating their degrees of exposure, *S* is the susceptibility of the SES, *C* is the capacity of the SES to cope, adapt and resist the hazard, *R* is the risk faced by the SES and *M*_*H*_ represents the characteristics of the multi-hazards (here intensity and frequency of droughts and floods). M_H_ represents the SES general exposure to the hazards under study. This conceptualization is in agreement with the IPCC summary report for policy makers ([2], p. 5), which defines risk as the *“potential for consequences*” where a valuable element is at stake and its outcome uncertain. This framework serves as a template for a reduced form of analysis allowing for the operationalization of the complex concept of vulnerability to a place based assessment. Note that all the quantities in Eq 1 are assessed by set of indicators which have been developed through participatory methods as described in Asare-Kyei *et al*. [13].

### 3.2 Participatory indicator development

Asare-Kyei *et al*. [13] followed a participatory approach to select indicators suitable for both quantitative and qualitative assessment of risks faced by people in West Africa under climate change. The methodology allowed for a representative participation of all stakeholder groups dealing with or affected by droughts and floods. Based on local stakeholder workshops, participants elicited indicators, which they considered as important in describing the risk they face. This revealed many new indicators, which were not or were rarely used in the literature related to West African risk assessment in the context of climate change.

A standardized questionnaire was developed to collect household’s fine scale data for each applicable indicator identified in Asare-Kyei *et al*. [13] in the three case studies. The selection of households was done with the use of a sampling frame received from the local authorities. The sampling frame contained information about communities frequently affected by floods and droughts, number of people affected, population as well as relief items provided by the local authorities. Almost all of the communities (over 90% in all study areas) frequently affected by the hazards were sampled. Within each community cluster, simple random sampling was used to select households usually affected by the hazards based on the sampling frame provided. The number selected from each community depended on total number of affected households, thus communities with higher affected populations received more representation. Unaffected households in these communities were also randomly selected to serve as basis for comparing the responses from affected households. In addition, 10 focus group discussions were held in the three study areas to capture the processes and impacts associated with droughts and floods and situations where the two hazards occurred in the same year. In the Vea study area, a total of 240 households were sampled and interviewed whilst 100 and 92 households were respectively sampled and interviewed in the Dano and Dassari study areas. The total number of households used in this study was therefore 432.

For indicators which cannot be described by household data such as Green Vegetation Cover, soil organic matter, population density, and others, secondary data were used. While some of these secondary data came from local statistical reports, some were also retrieved from remote sensing data and spatial analysis in a Geographic Information System (GIS). S1 Table in the supplementary information describes the construction of the data values for each indicator.

#### 3.2.1. Ethical statement regarding the use of household surveys/interviews

This study was approved and supported by UNU-EHS. The UNU-EHS, as a UN institution has the official mandate to conduct human subjects’ research specifically with regard to social vulnerability. The scientific committee responsible for this research is composed of senior researchers within the institute including the director, Prof. Dr. Jakob Rhyner, heads of various academic sections, Dr. Fabrice Renaud, Dr. Matthias Garschagen etc. It must be noted also that the human subject research conducted by UNU-EHS doesn’t involve clinical human experiments or samples but more simply of surveys and interviews for social vulnerability and disaster risk assessments. We apply rigorously basic principles: questionnaires are only filled in with approval of respondents; anonymity is strictly respected in assessing the results; no individual information is ever divulged; questionnaires are never shared.

At the start of each interview session, the objectives of the study were explained to the households and their verbal consent was sought. Written consent was not used because almost all the households sampled could neither read nor write and a request to make them thumbprint something they did not understand would have complicated the field survey. All the sampled households willingly and enthusiastically agreed to participate in the survey. Article preparation and submission protocol in place at UNU-EHS was followed and all research procedure was approved. Almost all the households’ heads or representatives who participated in the survey had their consent recorded. However, because the survey was conducted in remote, inaccessible communities, in less than 5% of cases, the recorder battery had run out and consent was taken in the presence of community key informants who acted as witnesses and supported the research.

### 3.3. Normalization and weighting of indicators

The re-scaling normalization technique was applied to convert different measurement units into a dimensionless unit. This method (Eq 3) normalizes indicators X to have an identical range between 0 and 1.

The drawback of this approach is that outliers can distort the transformed indicator. To prevent this, the exploratory data analysis described in the supporting information (S2 File) removed all extreme values (outliers) within the datasets based on expert knowledge. This rescaling normalization approach, however, has an advantage of widening the range of indicators lying within a small interval and increases the effect on the composite indicator more than the z-score transformation which has been used by Damm [14]. The world risk report used this approach to develop the “*World Risk Index*” [24,25].

After the indicators have been normalized, they were weighted using an expert opinion approach [14]. This approach allowed to better reflect policy priorities and the relevance of indicators for populations at risk to explain the risk and vulnerability in the study area. As explained in Asare-Kyei *et al*. [13], the experts provided rankings for all indicators within each vulnerability component. This ranking was converted to weights before the indicators were combined to develop the vulnerability index. The rank to weight conversion model developed by Al-Essa [43] was used in this study and assumes a linear relationship between ranks and weight.

For any set of *n* ranked indicators within a subcomponent and assuming a weight of 100% for the first-ranked (most important) indicator, the percentage weight of an indicator ranked as *r* can be derived by using the model developed by Al-Essa [43] and presented in Eq 2 in S2 File.

For details about this rank to weights conversion as applied in this study see Al-Essa [43], Stillwell *et al*. [44], Baron and Barrett [45] and Lootsma [46].

### 3.4. Aggregation of the composite vulnerability index

Applying the linear aggregation method, the normalized and weighted indicators were summed up to derive the composite vulnerability index. This approach has been applied in several studies such as Damm [14] in mapping socio-ecological vulnerability to flooding in Germany, and by Beck *et al*. [34], Birkmann *et al*. [25] and Welle *et al*. [24] in developing the World Risk Reports since 2011. Although there are other aggregation techniques, the linear aggregation technique proposed in this study is the most widespread aggregation method. This approach is basically the summation of weighted and normalized individual indicators.

This method imposes limitations on the nature of individual indicators. For example, to get a meaningful composite indicator (CI) is dependent on the quality of the underlying individual indicators and the measurement units. It also has implications for the interpretation of weights. This additive aggregation function works only if the individual indicators are mutually independent. This implies that the function allows the assessment of the marginal contribution of each indicator separately [47].

The linear aggregation technique applied in this study is given as:

$$CI_{c}=\sum_{q=1}^{Q}w_{q}I_{qc}$$ (3)

With $\sum_{q}w_{q}=1$ and $0\leq w_{q}\leq 1$ for all q=1,…,Q and c=1,…,M.

*C* is sub-component of vulnerability such as susceptibility, *M* is number of sub-components, *q* represents individual indicators, *W* is the weight applied to the indicator and Q is the number of indicators in a sub-component.

Using Eq 3, a three tier aggregation process was followed to develop the West Sudanian Community Vulnerability Index (WESCVI).

### 3.5 Developing the West Sudanian Community Vulnerability Index (WESCVI)

**Fig 4:**
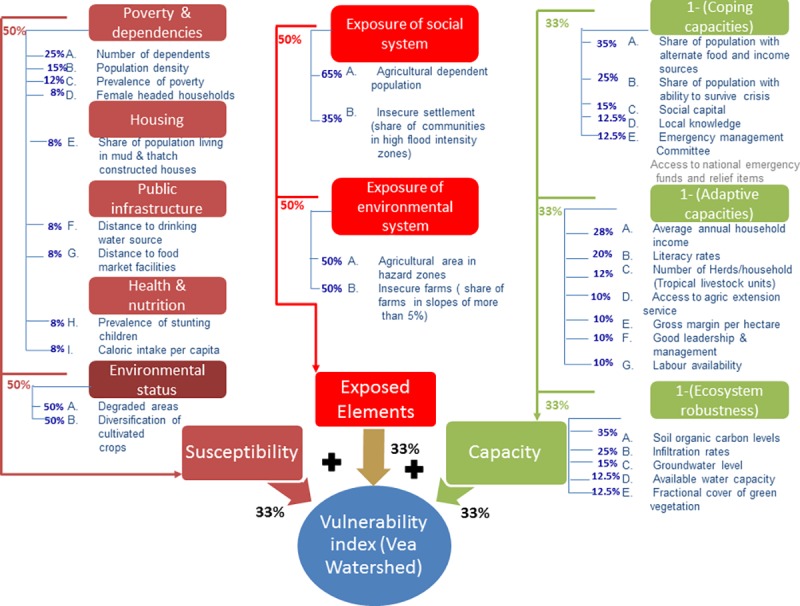
Schematic representation of the development of the West Sudanian Community Vulnerability Index (WESCVI) in the Vea study area of Ghana.

To quantify vulnerability means applying the weights to the data values of each variable and adding them up. Before doing so, a sub-index for each component was developed (see Fig 4).

As shown in Fig 4 for the Vea study area, the weight applied to each indicator is given in percentages. It must be noted that the indicators within each component have been listed in order of the ranking provided by the experts. The ranks for the first three or four indicators have been converted to weights as described above. For the exposed elements component, two indicators each for exposure of social system and ecological system exposure finally went to the computation of the exposure index after the bivariate correlation analysis (see Indicators A, B and A, B in Fig 4).

Note that Fig 4 and the corresponding figures in the supporting information (S1 Fig and S2 Fig) also illustrate the constituents of the community risk profiles. The figures show all the final components, sub-components and indicators that help to anticipate the level to which a community could be impacted by droughts, floods or a combination of the two hazards.

There are four thematic areas within the susceptibility component of the social subsystem according to which the indicators have been structured. These are ‘poverty and dependencies’, ‘housing conditions’, ‘public infrastructure’ and ‘health and nutrition’. The further categorization of the indicators into these thematic areas can allow for the development of additional sub-indices if so desired and thus will be crucial for determining which social aspect is most or least important in influencing the vulnerability of the people living in the study areas.

The capacity component has three sub-components: coping capacity, adaptive capacity and ecosystem robustness. An index was calculated for each of these sub-components by applying Eq 6 before being combined into the capacity index. Each of these sub-components were given equal weights of 33%, thus giving the social system a higher weight of 66% compared to the 33% from the ecological system. The reason is that capacity to cope or adapt is more construed to be pertaining to the social system than to the ecological system [25]. Weighting them equally here would mean underestimating the inherent ability of social systems to respond through coping and adaptation measures to the impact of the hazards.

It must be noted that in quantifying the WESCVI, coping capacities are not considered but instead their lack thereof. This lack of coping capacity is estimated by subtracting the estimated coping capacity value from one. This approach, which is also used in the estimation of the World Risk Index [24,25] was used to calculate lack of adaptive capacity and lack of ecosystem robustness. In vulnerability analysis, susceptibility by definition is construed to mean all factors that increase vulnerability whilst capacities do the opposite effect. Therefore, the negative variants of data values were used for susceptibility (e.g. distance of more than 30 minutes to water source) whilst positive variants of capacity indicators were used (e.g. literacy levels instead of illiteracy levels).

The WESCVI was finally estimated by combining the three indices describing exposed elements, susceptibility and (lack of) capacity. The vulnerability indices for the Dano (S1 Fig) and Dassari (S2 Fig) were estimated by using the same approach described above for the Vea study area. It must be noted that different set of indicators were used for each study area based on the results from Asare-Kyei *et al*. [13] and that this assessment in the present study is not meant for comparing the vulnerability or risk profiles of the different three study areas.

### 3.6 Multi-hazard index development

**Fig 5:**
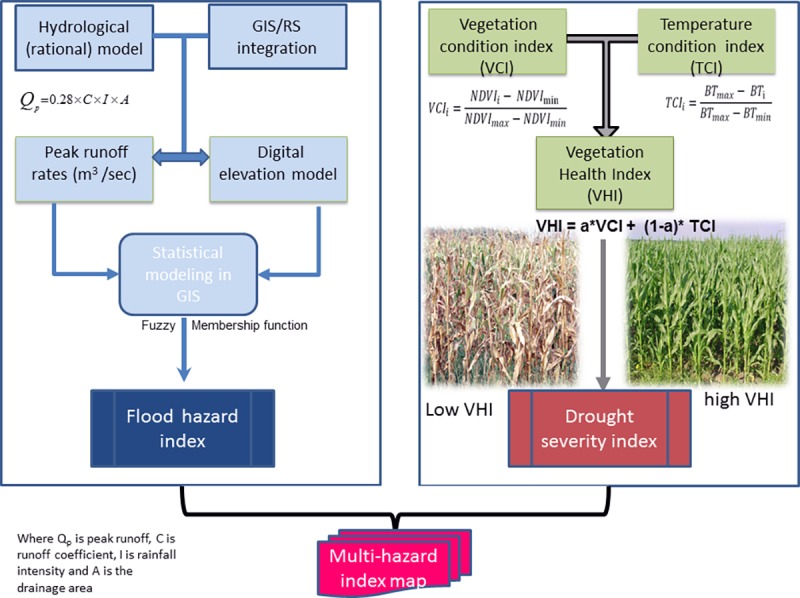
Development of multi-hazard index map. The figure on the left is a modified representation of the flood modelling approach introduced in Asare-Kyei *et al*. [38] whilst the figure on the right is a modified abstraction of FAO GIEWS [48] illustrating the development of DSI computed from the mean season of the VHI. VCI is the scaling of maximum and minimum Normalized Difference Vegetation Index (NDVI) and TCI is the scaling of maximum and minimum brightness temperature (BT), estimated from thermal infrared band of AVHRR channel 4 [49]. The final VHI is derived by applying weight, “a” to the VCI and TCI. The end results of these two methods were combined in GIS to develop the multi-hazard map.

The development of the multi-hazard index maps considered two components (see Fig 5), integrating the flood hazard intensity developed in Asare-Kyei *et al*. [16] and drought hazard. The first part was the development of a flood hazard index map. This approach presented in detail in Asare-Kyei *et al*., [38] drew on the strengths of a simple hydrological model and statistical methods integrated in GIS to develop a Flood Hazard Index (FHI) to an acceptable accuracy level. The FHI was validated with participatory GIS techniques using information provided by local disaster managers and historical data. The flood hazard component shows the intensity of flood at the pixel level on a scale of 1 to 5, with one being areas with least flood intensity and 5, areas of highest flood intensity.

The second component involves the development of drought hazard index termed the Drought Severity Index (DSI). From Fig 5, the DSI is computed from Vegetation Condition Index (VCI) and Temperature Condition Index (TCI) as explained in FAO GIEWS [48]. In this study, the final Vegetation Health Index (VHI) dataset was received from FAO Global Information and Early Warning System on Food and Agriculture (GIEWS) covering a period of 30 years (1984 to 2013). The mean VHI is an average of the decadal VHI values over the crop growing season to date and have non-cropland areas masked to cover only cultivated land. It is a good indicator of drought at the pixel level [48].

**Fig 6:**
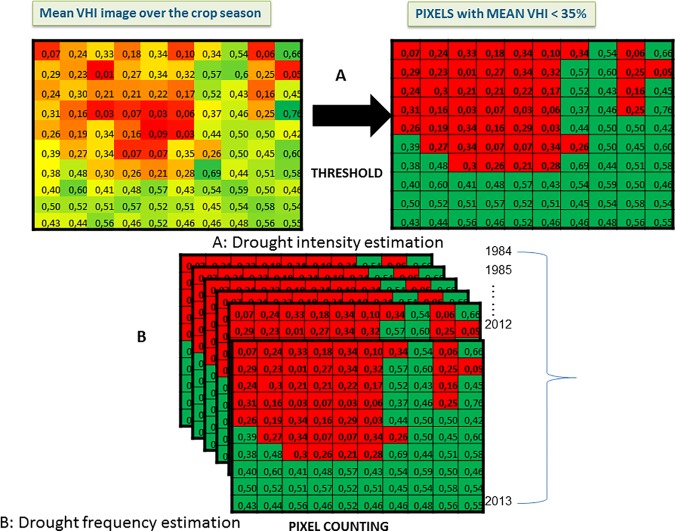
Estimating drought intensity and frequency over the study area. Conceptual basis for estimating the drought frequency over the 30-year period is from FAO GIEWS [48] and Rojas *et al*. [49].

The mean VHI which measures the drought intensity, was temporally integrated for every major season from 1984 to 2013 to derive the seasonal mean VHI. Two main estimations pathways were followed to derive the DSI which measures both the magnitude (intensity) of the drought and its frequency. The intensity was measured by computing the thirty-year average VHI (Fig 6A). Kogan [50] developed a threshold value of 35% below which a pixel is described as having agricultural drought condition. This threshold value was set by correlating VCI with different crop yields and various ecological conditions. The result was a logarithmic fit between VCI and crop yields at r-square of 0.79 [49,50].

To estimate the frequency of droughts at each pixel, a routine was established in the statistical software, R that calculates the number of times within the 30-year period that a pixel registers a VHI value of less than 35. Using this approach, the frequency of drought was established for every pixel over the entire study area (Fig 6B). The highest frequency was found to be 10 indicating that those pixels have registered exceptional drought conditions in 10 out of the 30-year period. Table 2 presents the classification of the drought frequency and intensity into five classes corresponding to the categories of the FHI.

**Table 2:** Classification of drought frequency and intensity datasets.

Frequency	Drought category	Mean VHI (intensity)	DSI at pixel level
**9–10**	Exceptional drought	<35	5
**7–8**	Extreme drought	36–45	4
**5–6**	Severe drought	46–55	3
**3–4**	Moderate drought	56–65	2
**1–2**	abnormal drought	66–75	1
**0**	no drought	>75	1

Classification according to the Jenks method implemented in ESRI ArcGIS and as modified from FAO GIEWS [48]. VHI is Vegetation Health Index and DSI is Drought Severity Index.

The drought frequency and intensity were normalized between 0 and 1 and combined using the weighted linear combination method given in Eq 7 [51] to produce the DSI in a GIS. The method permits the assignment of weights, which indicates the relative importance of a layer. The weights must sum up to one. In this study, the two standardized layers were considered equally important, thereby assigning a weight of 0.5 each to the layers in Eq (4).

$$DSI=\sum_{i=1}^{n}0.5X(av.VHI)+0.5X(\mathrm{droughtfreq})$$ (4)

Where i indicates the number of pixels or spatial units within each layer. This formulation then allowed the spatial combination of FHI and DSI to derive the multi-hazard index maps. Eq 7 was again applied to combine the DSI and FHI to derive the Multi-Hazard Index (M_H_I) map. It is important to mention that there are other approaches one could follow to combine the two hazards. Another example could be using the maximum function, in which case, a more than usual higher value in one quantity (hazard) could be rewarded. However, in this study, the weighted average function was found to be much simpler to implement. It therefore remains a possibility for subsequent studies to test the results of using different approaches of combining the two hazards. Note that the flood intensity (FHI) was also later normalized between 0 and 1 to allow for the spatial combination with the DSI.

### 3.7 Risk profile approaches

**Fig 7:**
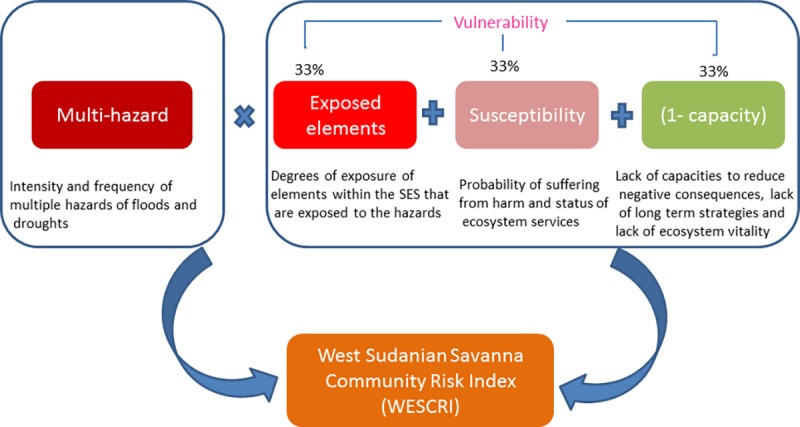
The modular structure of the WESCRI.

Once the vulnerability and multi-hazard indices are estimated, the multi-risk profiles of all the communities can be estimated by implementing Eq 2. Fig 7 shows how the derivation of the final risk profile of the communities in the study areas.

Populations exposed to the hazards were not intersected or overlaid with the quantity, M_H_ as this was already captured in the vulnerability estimation pathway where the degrees of exposure of the critical elements (people, farmlands, protected area etc.) were used. The quantity, M_H_ measures a spatially explicit assessment of the SES general exposure to the two hazards of floods and drought.

### 3.8 Validation of risk and vulnerability indices

The robustness and the quality of the composite vulnerability indicator as well as the soundness of the risk profiles in estimating the potential impacts of the hazards on the communities studied were further tested. In this study, two main approaches are presented to evaluate the results of the community level vulnerability and risk indices.

#### 3.8.1 The concept of community impact score

A novel technique is introduced in this study to validate the underlying models and assumptions used to develop the community risk profiles with real historical impact data collected from at risk populations. To do this type of risk model validation, which as far as available literature on risk assessment confirms has not been pursued, an approach to develop an impact score for each community cluster called ‘community impact score’ (CIS) is introduced. The CIS measures the cumulative impact of the occurrence of the multiple hazards over a period of five years. During the field work as described above, households were asked to recount the impact they had suffered over the last five years as result of the occurrence of drought, floods and multiple hazard occurrence. The impact assessment captured data on the following key variables.

- Population affected by floods (%) by community cluster
- Population affected by droughts (%) by community cluster
- Population affected by floods and droughts in the same year (%) by community cluster
- Average area of cropland affected per community (acres)
- Average number of livestock affected/killed by hazards
- Number of people killed by floods (human loss)
- Number of housing units destroyed or partially damaged by floods
- Economic value of properties (houses, personal effects etc.) destroyed by floods or fires occasioned by prolonged drought.

The results of this detailed assessment are presented in the supporting information (S2 Table). To develop the CIS, these impact variables were first standardized to make any combination meaningful. The linear interpolation method was applied to standardize the impact variables. This procedure results in standardized impact values on a scale of 1 to 4; with one being the lowest impact level and 4 the category with the highest impact level. The linear interpolation scheme (Eq 5) as applied in Morjani [52] was used to standardize all the variables. This procedure first involves the determination of minimum and maximum impact levels and then calculating the slope and intercepts of the impact level for each variable. The minimum and maximum values were used as the known variables in the horizontal axis whilst the scale range from 1 to 4 was used as the known variables in the vertical axis in the estimation of the slope and intercept. The resulting slope and intercept values of the respective variables were then applied to each impact variable value using Eq 5 below. This procedure resulted in standardized impact variables, which were then multiplied to derive the CIS.

$$IV_{st}=\mathrm{Integer}\left(\left[\mathrm{slope}\times IV\right]+\mathrm{int}+0.5\right)$$ (5)

Where *IV* is the impact variable, *IV*_*st*_ is the standardized impact variable and “int” is the intercept. The derived CIS was then scaled between 0 and 1 to correspond to the multi-risk index. Two statistical model validation tools were used to assess how well the risk model approximate actual disaster impacts. The Root Mean Square Error (RMSE) and the Coefficient of determination (r2) [53,54] were used.

#### 3.8.2 Sensitivity analysis

The sensitivity of the vulnerability model was analyzed by examining the sources of variation in the model output to determine the contribution of the input variables to this variation. The study favored the use of local sensitivity analysis, which allows the influence of one varying variable to be studied while all the other variables are held constant. A local sensitivity analysis could reveal complementary information that has policy relevance, allowing policy makers to understand the variables which when intervened, could have significant impact on the overall vulnerability of the communities [25]. This is important for the objective of this study which seeks to identify variables contributing to household’s vulnerability and risk and to support programmatic interventions at the community level. In this study, sensitivity was analyzed by way of volatility of the variable to be changed in relation to its original state. In accordance with Damm [14], OECD [47] and Groh *et al*. [55], volatility is measured by the standard deviation of community vulnerability index across all community clusters in each study area.

## 4. Results and discussion

The results and discussion for all the sub-components are presented in the supporting information (S3 File), where exposure, susceptibility and capacity are separately discussed. Also in S3 File, tables showing the community rankings for all sub-components are presented and discussed. Exposure is presented in Table A of S3 File, susceptibility rankings in Table B of S3 File and lack of capacity is presented in Table C of S3 File.

### 4.1. The West Sudanian Community Vulnerability Index (WESCVI)

**Fig 8:**
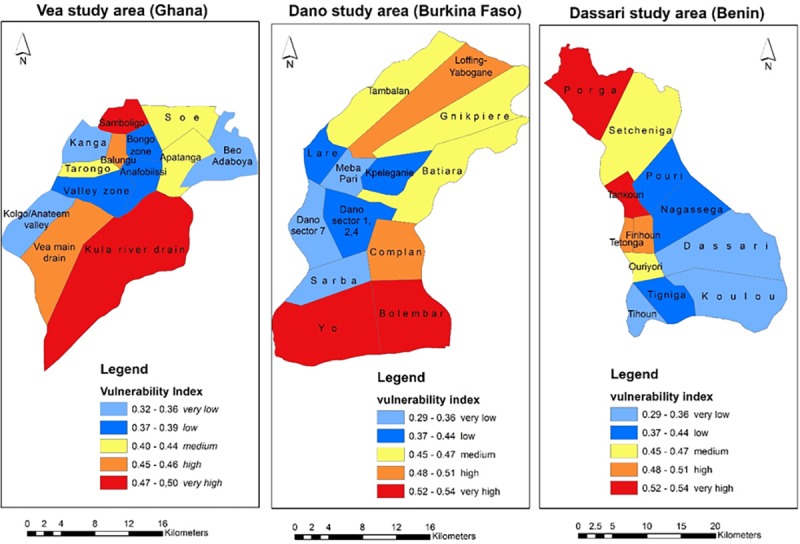
The Composite community vulnerability index. Note that the class ranges for the three maps differ because each represents a distinct study area. The vulnerability indices for the study areas are presented together here just to conserve space and they are not intended for comparisons.

Following the three tier-aggregation procedures, the sub-indices of exposure, susceptibility and lack of capacity were combined to develop the composite vulnerability index and mapped in GIS (Fig 8). This composite index measures the degree of vulnerability across all community clusters in the study areas. To illustrate the variability of vulnerability across the clusters, five classes of vulnerability have been developed using the Quantile classification method. The classes range from 1, for lowest vulnerability level to 5, for highest vulnerability level. The same classification method was used for all the vulnerability sub-components of exposure, susceptibility and capacity, which explains the different value ranges of the classes between study sites.

Results show that in the Vea study area, the Samboligo community cluster is the most vulnerable area with a vulnerability score of 0.50. It is followed by communities in the Kula River drain (0.48) and Balungu (0.46). In this context, the level of exposure of these communities explains the high vulnerability. For instance, although the Kula River communities have the highest capacity to cope and adapt to changing climate patterns (see Table C in S3 File) and relatively moderate level of susceptibility, its high level of exposure (Table A in S3 File) affects its overall vulnerability score. In the case of Samboligo, high levels of susceptibility and relatively low capacity to cope and adapt make it highly vulnerable even though its exposure to the hazards is relatively much lower. Balungu’s high vulnerability status results from moderate to high level scores recorded for all three vulnerability components. It has moderate levels of vulnerability rankings of 4, 3 and 5 out of 13 community clusters for exposure, susceptibility and lack of capacity, respectively. This means that in vulnerability analysis, a consistent moderate ranking of an area or system will ultimately put the community or system into a high vulnerability class. In the Vea area, Samboligo emerges as the hotspot of vulnerability due its lowest level of coping capacity, poor adaptive capacity and generally poor state of its ecosystem. It is also highly susceptible to droughts and floods as results of inherent poverty and high dependency ratios, poor housing and lack of infrastructure. The results of the household survey show, that as much as 93% of its inhabitants have poor housing conditions living in primarily mud and thatch houses which are easily damaged by flash floods and torrential rains. On the other hand, the Beo-Adaboya, Kolgo Anateem and Kanga are clusters with the least vulnerable levels. In the Kanga area, moderate levels of susceptibility are mitigated by low exposure (0.13 in Table A in S3 File), high coping and adaptive capacities and generally robust ecosystems.

In the Dano study area, the hotspots of vulnerability are the Yo, Bolembar and Loffing-Yabogane community clusters. The Yo area remains the highest vulnerable area due its high susceptibility to the hazards and weak capacities. It also has a moderate exposure ranking of 5 out of 13 clusters. The vulnerability of the communities in the Yo cluster results mainly from its low levels of coping and adaptive capacities. Only 37% of its inhabitants have adequate local knowledge regarding droughts and floods coping strategies, DRR measures, etc. This coupled with a meager percentage of households having access to alternate food and income sources (12.5%) and an absolute illiteracy level makes the Yo area a hotspot of vulnerability in the commune of Dano in Burkina Faso.

In the Dassari study area, Porga, Tankouri and Firihoun are the three top vulnerability hotspots with Tihoun, Dassari and Koulou being the least vulnerable areas. The high levels of exposure in the Porga area counteracts its moderate levels of susceptibility and capacity, making it the most vulnerable area in the Dassari arrondissement of Benin. This high exposure results primarily from two indicators, ‘insecure settlement’ and ‘agricultural area in hazard zones’. All the settlements in the area (100%) are located in high flood and drought intensity zones whilst over 33% of their agricultural land is also found in high flood intensity zone. The study revealed frequent destruction of settlements by wild fires due to prolonged drought conditions and also by flash floods. As much as 90% of all houses are made of mud and thatch and are of poor quality. These houses are hastily constructed after each disaster. These settlements may be inexpensive to build but are more physically vulnerable to hazards such as floods and increase the risk to physical injury to those who live in them [56].

### 4.2 Risk profiles from multiple hazards

By combining the vulnerability and the multi-hazard indices through the arithmetic multiplicative function in GIS (Eq 2), the multi-risk profiles of all communities in the study area were quantified in line with our research objective. This multi-risk profile represents the combined effect of the occurrence of multiple hazards and their interaction with vulnerable SES. It measures the extent to which households within the communities will be impacted by floods, droughts and a combination of them.

**Fig 9:**
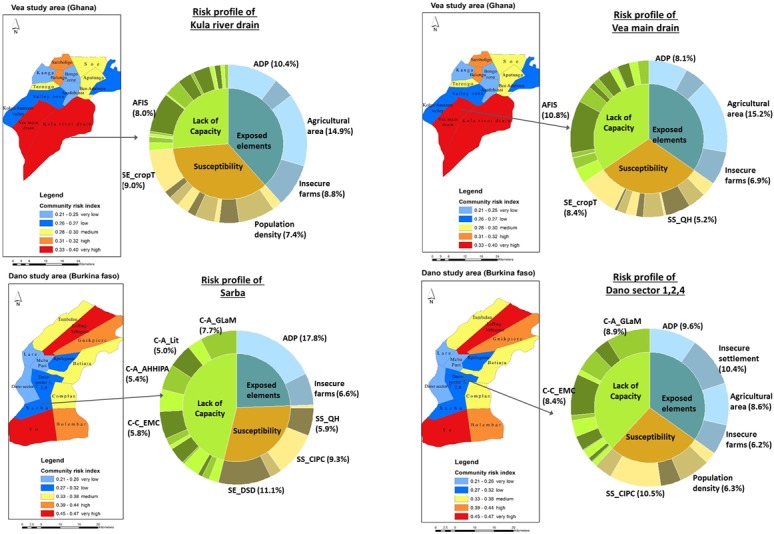
The Risk profiles of two community clusters in the Vea and Dano study area. Following the approach in the World Risk Index [25,34], the risk indices have been translated into five qualitative classification scheme of very high (5), high (4), medium (3), low (2) and very low (1). Classification algorithm employed is the Quantile method. In this figure, two levels of factors contributing to final community risk are presented. The first is the three major components of risk, which are exposure, susceptibility and lack of capacity. The second level shows the relative contribution of each indicator to first, the sub-component such as exposure and then to final risk. Only indicators contributing to more than 5% of the final risk are shown. Major contributory factors to risk are: AFIS = access to alternative food and income sources; SE-CropT = crop type or the proxy of crop diversification practices; ADP = agricultural dependent population; SS-QH = quality of housing; SE-DSD = length of dry season duration; CC-EMC = presence of emergency management committee; C-A AHHIPA = annual household income; CA-Lit = levels of adult population above age 15; CA-GLaM = good leadership and management at the community level and CIPC = caloric intake per capita.

In Fig 9, the result of the WESCRI is presented and shows contrasting levels of risk among community clusters.

**Fig 10:**
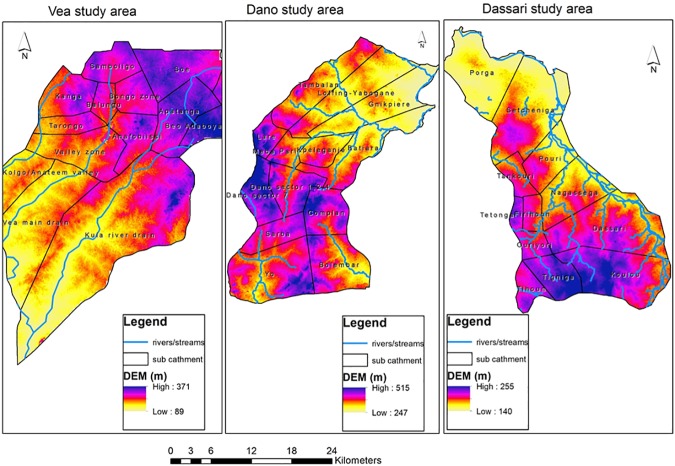
Digital Elevation model of the three study areas (From Asare-Kyei *et al*. [16].

Also presented in Fig 10 is a Digital Elevation Model (DEM) of the three study areas showing that low lying areas generally exhibit high total risk to the two hazards.

In the Vea study area, the Kula River drain and Vea Main drain remain the hotspot of risk to droughts and floods. Communities in these areas are characterized by high exposure to floods [16] and droughts and at the same time have the highest levels of vulnerability. The study shows the strong effect of exposure to hazards have on the overall risk faced by a community. This is evident from the relatively good scores recorded by the two clusters in the vulnerability sub-components of susceptibility and capacity to cope, adapt and state of ecosystem.

Kula River drain in particular has the highest capacity in the Vea area, yet it has the highest vulnerability and subsequently is amongst the high risk areas due primarily to its exposure to floods and droughts. This means that an area will still be classified as having significantly high multiple risk levels when unusually high exposure levels are combined with moderate levels of susceptibility, no matter how strong its capacity to cope and adapt to the hazards might be. The reverse is also true as poor state of inherent conditions and lack of capacity could still place an area at high risk although its exposure to the hazards is low. This is the case of Samboligo where its low exposure index of 0.297 does not mitigate the high negative scores in susceptibility (0.594) and lack of capacity (0.614). Balungu cluster of communities shows reverse situation where high levels of vulnerability (Fig 8) are compensated by very low levels of multiple hazards occurrence. Therefore, we need the detailed knowledge of the communities’ specific risk profiles to adjust risk prevention and adaptation measures that may be available in the locality.

In Fig 9, the detail risk profiles of two community clusters each in the Vea and Dano study areas are presented and show the main causative factors of risk in the area. In the Vea study area, the two community clusters all fall into the high risk index category and a look into the indicators contributing to this high risk class show that both clusters have similar underlying risk profiles. In both cases, exposed elements is the highest causative factor to total risk, contributing 38.3% in the Kula River drain cluster and 34.7% in the Vea main drain cluster. Although these areas have moderate susceptibility levels, they fall into high risk category as a result of the extremely high exposure levels (Fig 9). There are also similar profiles at the sub-component level, exposed elements in both clusters are more influenced by agriculture area in hazard zones, agricultural dependent population (ADP) and insecure farms whilst Alternate Food and Income Sources (AFIS) is the main cause of communities’ lack of capacity. However, the Dano community clusters present different risk profiles. Although both clusters, Sarba and Dano sector 1,2,4 fall in a low risk category, their risk profiles are markedly different from each other. Exposed elements contribute far less to risk (24.4%) in the Sarba area and far more to risk in the Dano sector (34.8%). Whilst three indicators, dry season duration (DSD), caloric intake per capita (CIPC) and housing are the main drivers of susceptibility in the Sarba cluster, only CIPC and population density have a significant contribution to susceptibility in the Dano sector 1,2,4 cluster. These results show that different communities can be part of the same risk category, but the underlying factors defining their risk levels can be fundamentally different from each other. It is therefore incumbent on policy makers and practitioners to understand the detail causative factors of risk to deploy interventions that effectively targets the principal factors affecting risk in a given area.

In the Dano study area, Yo, Loffing-Yabogane as well as Bolember and Gnipiere are the hotspots of risk. These areas are also the hotspots of vulnerability. However, in the Complan community cluster, vulnerability is comparatively lower because of low levels of multiple hazards occurrences pushing the communities in the area into a medium risk class. The high level of risk in these community clusters are due to underlying poor socio-economic conditions. Only 37% of its inhabitants have adequate local knowledge regarding droughts and floods coping strategies, DRR measures etc. This coupled with a small percentage having access to alternate food and income sources (12.5%) and an absolute illiteracy level in most clusters (100%) makes the area a hotspot of vulnerability and risk.

In the Dassari study area, Porga, Sétchindiga followed by Dassari and Tankouri are the risk hotspots. The medium vulnerability profile of Sétchindiga was not enough to mitigate the effects of high multiple hazards occurrence and, as can be seen in Fig 9, pushes the communities in the area to high risk levels. Similarly, Dassari has a significant lower level of vulnerability (Fig 8), yet high occurrence of multiple hazards eventually increases its overall risk to droughts and floods.

Maximum risk level for all community clusters studied is in the Yo area of Dano whilst the Meba Pari community clusters have the least risk levels. Also, communities in the Kula River drain registered significant high risk. The statistically significant high risk faced by people in the Dano area results from poor socio-economic systems, high exposure to droughts and rainstorms. The household survey found several cases of collapsed buildings due to flash floods and generally poor living standards as evident in the high vulnerability scores estimated.

### 4.3 Results and discussion of the CIS validation concept

**Fig 11:**
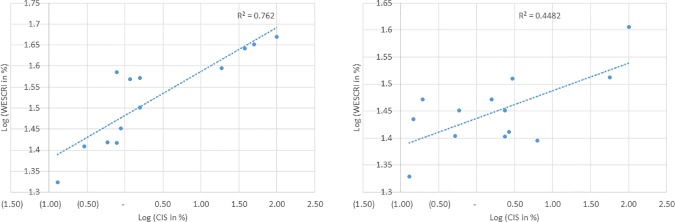
Relationship between simulated risk (WESCRI) and observed disaster impacts (CIS). Left chart represents the Vea study area with the trendline below: LogWESCRI=0.1045×LogCIS+1.4828 Right chart shows the Dano study area with the trendline below: LogWESCRI=0.0511×LogCIS+1.4367

The CIS estimated above was compared with the simulated risk index to determine the robustness of modelling procedures. In the Vea study area, the RMSE of the estimated WESCRI was relatively low at 0.29, R2 was found to be 0.45. In the Dano study, RMSE of the estimated WESCRI was also found to be 0.29, R2 was estimated at 0.76. The RMSE was lower for both study areas indicating that the multi-risk model closely approximates the observed impacts of the hazards. In the Dano study area, as much as 76% of the variance in observed impact of hazards was explained by the risk model whilst 45% of the variability in observed hazard impact was explained in the Vea study area by the multi-risk modelling procedures (Fig 11).

These levels of variance are considered relatively high against the background of uncertainties associated with the observed impact data. The impact data as recounted by at risk populations were derived from memory and there were no systematically documented records of the impacts of the hazards. Most of the respondents were able to recount only the high intensity or magnitudes of the hazards and small impact events were generally not recalled. In the Dassari study area, the responses were found to be highly inconsistent and were subsequently discarded. Therefore, no validation based on reported impacts was possible. Fig 11 shows the strong linear relationship between the observed disaster impact and the modelled output of multi-risk index. As can be seen from Fig 11, despite the difficulties in recounting disaster impacts from memory, communities with high simulated disaster risk generally experienced high observed disaster impacts. This shows the vulnerability and risk models can generally be used in predicting high and low risk areas in the study areas with reasonable error margin.

### 4.4 Sensitivity analysis

In this study, six scenarios based on observed relationships between the input variables (indicators) and the vulnerability composites were carried out to understand which inputs accounted more to a community’s vulnerability profile. Table 3 presents the mean volatility of the six different scenarios compared to the original vulnerability estimations. In the Vea study area, volatility ranged from 0.05 to 0.06. Overall, the mean volatilities for all three study areas are found to be very low indicating that the sensitivity of the composite vulnerability index to the varied or excluded indicator is negligibly low. This means that the aggregation technique introduced, the weighting system applied as well as the modelling procedure followed resulted in robust estimates and that the final indices are largely unaffected by changes in single indicators. Similar results were found by Damm [14] in mapping flood risk in Germany.

**Table 3:** Mean volatility between 6 different vulnerability scenarios.

No.	Scenario	Mean volatility		
		Vea	Dano	Dassari
**1**	Equal weights of all indicators	0.050	0.071	0.048
**2**	Excluding Agricultural Dependent population	0.046	0.075	0.036
**3**	Excluding insecure settlement, population density, Soil organic carbon (Basfonds for Dano), Ability to survive crisis (alternate food % income source for Dano) and access to extension	0.049	0.051	0.036
**4**	Increased Agricultural Dependent population by 10%	0.056	0.074	0.043
**5**	A. Increased by 10% Agriculture area, population density, Caloric Intake per Capita and B. decrease by 10% SOC (Bas fonds in Dano & Dassari) and annual household income	0.057	0.076	0.043
**6**	Excluding number of dependents (Dano & Dassari, Vea) and distance to market (Vea)	0.047	0.066	0.039
	Minimum	0.046	0.051	0.036
	Maximum	0.057	0.076	0.048

## 5.0. Conclusions

The aim of this study was to carry out a multi-hazard risk assessment to floods and droughts using a bottom-up participatory process at the community level to derive community risk profiles and to develop a new concept for quantitative validation of risk assessment. The study analyzed a coupled SES based on three sets of indicators for the three case studies that have been verified and ranked by at risk population and local stakeholders. The study quantifies vulnerability and risk with the aim to support practitioners and policy makers with detailed information about vulnerability and risk profiles at the community level. This aspect of identifying high risk communities by mapping risk hotspots in the study areas is particularly relevant for practitioners and policy makers.

The study found that exposed elements are directly related to the pattern of flood and drought hazard intensities and consequently are key determinants of vulnerability. Besides the proximity to hazards, a major driving factor influencing community exposure is the indicator measuring the share of the population engaged in agriculture. This finding confirms the assertions by Adger *et al*. [56] and O’Brien *et al*. [57] that high Agricultural Dependent Population (ADP) means that a higher percentage of people are exposed to a climate sensitive sector of agriculture. In the study areas, rain-fed agriculture predominates [13] further aggravating people’s exposure to irregular rainfall. High ADP suggest lack of other employment options and therefore in the event of crop failures, farmers and their dependents have few opportunities to earn additional income [56,57].

The study found that an area will still be classified as having significantly high risk levels when unusually high exposure levels are combined with moderate levels of susceptibility, no matter how strong its capacity to cope and adapt to the hazards might be, (see Fig 9, particularly, Vea main drain and Kula clusters). The reverse is also true. However, poor state of inherent conditions and lack of total capacity could still place an area in high vulnerability zone although its exposure to the hazards is low. Therefore, it is very critical to understand the composition of factors contributing to the overall risk for the design of appropriate and adjusted disaster risk reduction measures.

Using five-year historical impact data collected from at risk populations, a novel technique was introduced to validate the underlying models and assumptions used to construct the risk profiles. The concept of CIS was thus introduced and measures the cumulative impact of multiple hazards in the study areas. Against the background of large uncertainties associated with the observed impact data, this study found relatively high levels of variance explained, 76% for the Dano study area and 45% for the Vea study area.

The results of the local sensitivity analysis show that the mean volatilities for all three study areas were very low; ranging from a low of 0.036 to a high of 0.076 indicating that the composite indicator is largely stable. This kind of local sensitivity analysis is useful for understanding the relative importance of the changed or varied indicator, an analysis which has implications for policy makers to understand the variables which when intervened upon, could affect the vulnerability index. For instance, the risk profiles shown in Fig 9 showed that varying agricultural areas in hazard zones in two community clusters (Kula river drain and Vea main drain) will have significant effect in the level of vulnerability and overall risk faced by the SES in those areas. Policy makers could therefore implement interventions aimed at reducing cropland area in high hazard zones.

In an attempt to deal with the on-going scientific debate on whether or not to include the exposure component in vulnerability assessment, this study provided an alternative approach where the degrees of exposure of elements in the SES (spatial dimension of exposure) are considered as contributing to the SES total vulnerability, rather than using the SES’s general exposure as part of vulnerability or rather than excluding the exposure term altogether. This procedure therefore eliminates a key drawback of the summation conceptualization of vulnerability which could place a community in a high vulnerability class although its exposure may be zero.

The point is that, in reality, people are still vulnerable even though they may not be exposed to any obvious hazard due to inherent depressed socio-economic conditions and intersection of its elements with some hazards that may not be too apparent to the people. However, even in the face of obvious lack of physical hazards, elements within the SES such as its farmlands, protected areas etc. could still be exposed, albeit partially or remotely due to cross scale interactions. This phenomenon is very common in the study areas where existing socio-economic conditions in most cases is very dire and leaves people vulnerable even though there are no obvious physical exposure. In the final risk assessment, however, where there is no SES general exposure, risk will be zero even though vulnerability could be high. This is the upside of the multiplicative effect which was finally used to estimate the risk index. This area of risk assessment where a system could still be vulnerable even though there may not be obvious linkages to physical hazards requires further studies.

The study provides a framework for conducting risk assessment for multiple cultural and social contexts spanning three countries using indicators developed from a bottom-up participatory process (see S3 Table). Unlike risk assessment from classical approaches, the differential risks from these three study areas therefore uniquely represents actual risks faced by its SES as identified by the at risk populations. At the same time, the study sets the pathway for conducting risk assessment using a unified indicator set if so desired by practitioners or policy makers. It must be noted however that, practitioners or policy makers desiring to conduct multiple hazard risk assessment based on the methodologies presented in this study need to have several scientific competencies to be able to follow all the approaches outlined here.

Studying risk profiles of rural communities also provides an insight on how to situate vulnerability, risk and climate change adaptation efforts within the context of the community’s sustainable development agenda and can help to develop appropriate, inclusive and well integrated mitigation and adaptation plans at the local level. To cope with climate change and achieve poverty reduction, it is essential to pursue actions at sector and community levels [58] and we believe the present study contributes greatly to efforts in this direction. Another key output is development of comprehensive methods allowing practitioners to conduct similar community level assessment and to continue to update the risk profiles. Generally, vulnerability and risk assessment are rarely verified against impact data. This is because such impact data are rarely available in the level of detail and/or spatial scale required. We attempted here to validate the computed risks by introducing the novel and pioneering concept of CIS which remains improvable but can allow for a first estimation of the validity of risk indices in global scientific studies of climate risk assessment.

## Supporting information

S1 File Background to natural hazards in the study areas. (PDF)

S2 File Exploratory data analysis and bivariate correlation analysis. (PDF)

S3 File Results of the main components of vulnerability and risk. (PDF)

S1 Fig Development of vulnerability index in the Dano study area. (TIF)

S2 Fig Development of vulnerability index in the Dassari study area. (TIF)

S1 Table Construction of data values of indicators and sources of data. (PDF)

S2 Table Variables used to develop the community impact score (PDF)

S3 Table Indicator reference table for West African risk assessment. (PDF)
