# Supplementary material for: Development and validation of risk profiles of West African rural communities facing multiple natural hazards
Source: PLoS One. 2017 Mar 1;12(3):e0171921. doi: 10.1371/journal.pone.0171921 (PMC5382969; doi:10.1371/journal.pone.0171921)
Supplement: S1 File — (PDF) [file pone.0171921.s001.pdf]

S.1. Text: Background to natural hazards in the study areas

S.1.1. The Vea study area

The Vea area (Fig. A) cuts across two districts in Ghana - Bolgatanga and Bongo - and covers an area of 1037.8 km^2. The city of Bolgatanga, the capital of Upper East region is found in this area. This study site is the most urbanized of the three study areas and has a well-developed road network, schools, market access, hospitals, irrigation dams and electricity. Consequently, it has a relatively higher population density of about 104 persons per km^2. Hydrologically, it falls within the White Volta sub-basin, which extends from northern Ghana to mid Burkina Faso.

The area ranks high amongst areas most exposed to multiple natural hazards occasioned by climate variability. Similar to other parts of WA, studies have shown that this area experiences high variability in climate and hydrological flows [1,2]. According to Oduro-Afriyie and Adukpo [3], the area has frequently experienced floods in the past: between 1991 and 2013, the area has experienced eight major floods, the largest number of people affected being in 1991 [4]. From 2007 to 2013, there have been consecutive flood events.

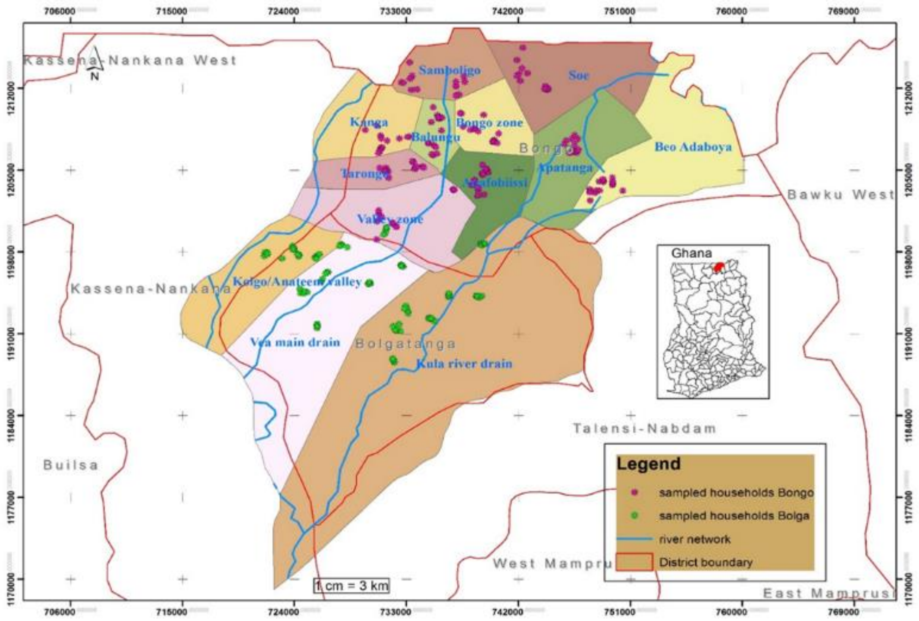

Fig. A. The Vea study area of Ghana (source: Asare-Kyei *et al.* [5]

In 2007, floods followed immediately after a long period of drought and damaged the initial cereal harvest. During this flood disaster, at least 20 people died and an estimated 400,000 people were affected, over 90,000 people were displaced and nearly 20,000 homes were damaged [6]. The long-term and economic impacts on the northern regional economy are still not known but the World Bank [7], estimated the damage to be around US$130 million. Ghana's National Disaster Management Organization (NADMO) reports that within a period of three years (2010 to 2012), a total of 702,204 people have been affected by floods in Northern Ghana, of which 42% are in the

Upper East region where the Vea study area is located. Within this same period, floods have killed 145 people, destroyed 72,391 houses and inundated 31,263.84 hectares of cropland. Of the 20,403 people affected by floods in the Upper East region in 2011, more than 54% were from the Bolgatanga and Bongo districts and virtually all the 3,428 houses that collapsed during this flood event were from the two districts that make up the study area.

It is estimated that 35% of the land area in Ghana (roughly 83,489 km2) is prone to drought, with the Sudanian Savanna zone facing the greatest hazards. Drought and their attendants' desertification is said to be advancing inland at an estimated 20,000 hectares per year [8], with its concomitant destruction of farmlands and livelihoods. The major drought event in recent times was in 1983 where over 12.5million people were affected, most of them located in the Sudanian Savanna zone. As much as 76.9% of all people affected by any disaster in Ghana are due to drought[1].

S.1.2. The Dano study area

The Dano study area (Fig. B) is essentially the third sub-administrative level in the province of Ioba of Burkina Faso and has an area of 633.8 km^2. Population density in this study area is about 59 persons per km^2. Hydrologically, it falls within the Black Volta sub-basin system, which forms the western part of the Volta basin.

[1] http://www.preventionweb.net/english/countries/statistics/?cid=67. Retrieved March, 5, 2013-

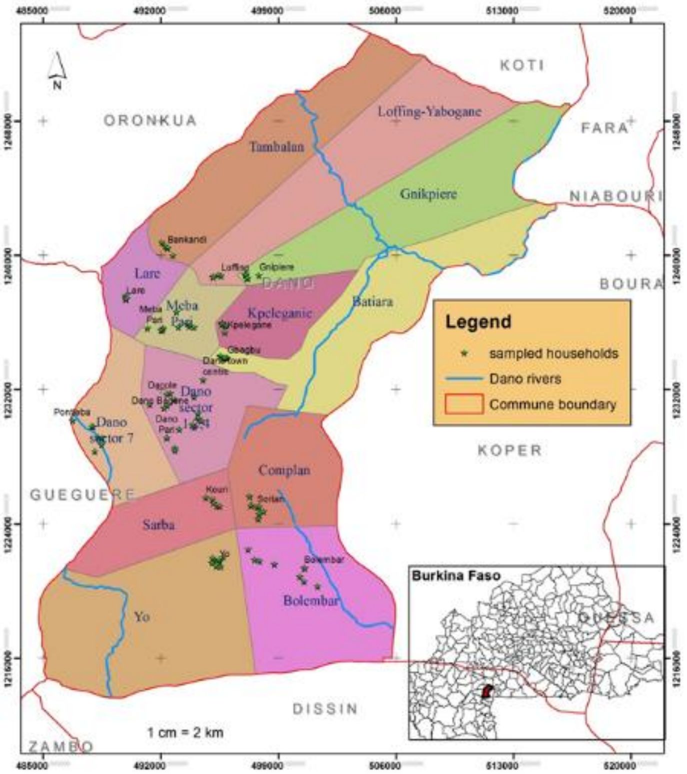

Fig. B. The Dano study area of Burkina Faso,source: Asare-Kyei *et al*. [5]

Compared to the Vea study area, local scale data on flood hazards in this area is limited and CONASUR, Burkina's disaster management organization believes Dano is not among the high flood zones in Burkina Faso. However, available records suggest that in 2009 heavy rains in Burkina Faso caused flooding in many parts of the country and forced officials to open the main gate of a hydroelectric dam (Bagre dam) which also caused flooding in downstream areas in northern Ghana [9]. During this flood, Burkina's main hospital was closed down. Whereas annual rainfall in Burkina Faso has been averaging 1,200 mm, as much as 300 mm occurred within one hour on September 1, 2009 and the Burkinabe Government estimated that it was going to cost US$152 million to face the consequences of the flooding. The Dano study area has severely been affected by floods from torrential rains. In 2008, 41 people were affected, another 493 inhabitants in 2012 were affected in Dano. The floods in 2010 in particular affected 12 other provinces where more than 160,000 people were directly affected and 14 people were reported dead.

Villages were devastated with damage to shelters, livestock, properties, agricultural fields, roads and wells [10]. The occurrence of flood in the area has been increasing in recent years. Field observations showed that most houses were easily damaged by flash floods caused by torrential rains. This phenomenon is a great source of worry for many households who already have to face daunting climate variability issues.

The United Nations office for Disaster Risk reduction (UNISDR) reports[2] in its database that, major drought events have occurred in Burkina Faso. Drought affected over 2.6million people in 1990, over 1.2million in 1980, 200,000 in 1988 and over 75,000 in 1995. The most severe drought event in recent times is the one in 2011 which caused the United Nations to organize an emergency meeting in Rome in a bid to avoid famine in the country. UNSIDR reports that the probability of drought occurring in Burkina Faso for a typical year is 0.19 and accounts for 84.8% of all people affected by any disaster in the country.

S.1.3. The Dassari study area

The Dassari study area (Fig. C) which covers an area of 657.1 km^2 falls in the third sub-national administrative level in Benin (known as the Arrondissement of Dassari) and has a population density of about 56 persons per km^2. In terms of hydrology, the study area falls within the Oti sub-basin of the Volta basin. The north-eastern corner of the study area forms part of the Pendjari National Park in West Africa.

At the national level, the worst flood in Benin since 1963 occurred in September 2010 when heavy downpour and influx from the Niger River flooded 55 out of the 77 municipalities in the country including the Materi commune where this study area is located. In this flood alone, over 680,000 people were affected, 800 cases of cholera were reported, 55,000 homes were destroyed and at least 56 people were killed [9]. It must be mentioned however, that, the frequent flooding from the Niger River affect the neighboring communities more than it does affect the Materi commune.

Similar to Dano, there is limited local scale data available on flood hazards in this study area. However available data suggest the area has also not been spared of the hydrological hazards that have affected the other two study areas. In 2010, 40 ha of crops were lost to floods in the commune Materi, in which the arrondissement of Dassari is located. In 2013, the local agricultural department had to distribute seeds to over 3600 farmers in the study area to replant about 223 ha which had been destroyed by floods in the previous farming season.

[2] http://www.preventionweb.net/english/countries/statistics/?cid=19: retrieved March 4, 2013

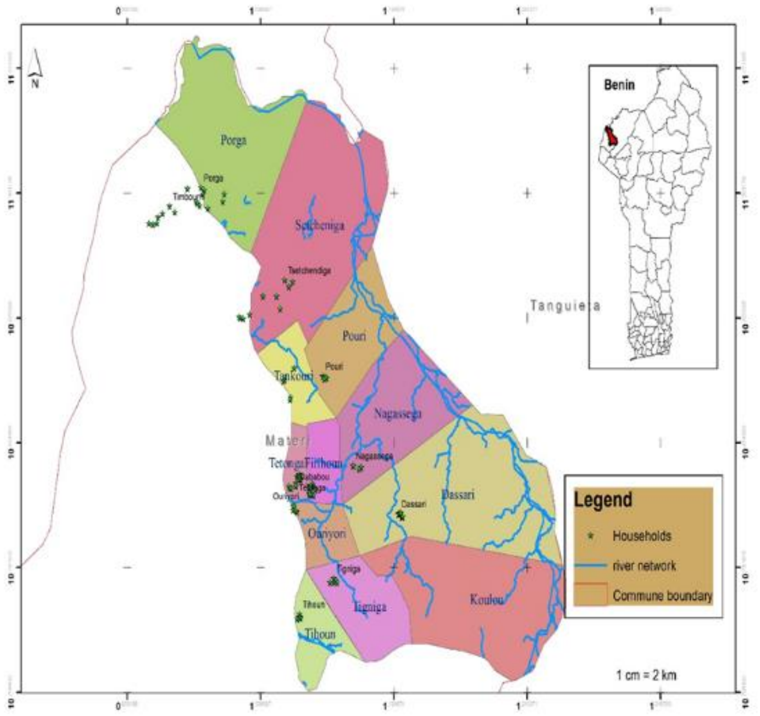

Fig. C. The Dassari study are of Benin. Asare-Kyei *et al.* [5]

The worst drought event to hit Benin in recent times is the one 1983 where over 2.1million people were affected and faced severe famine. Droughts alone accounts for 40.2% of people affected by any type of disaster in the country.

Drought has had a devastating impact on this ecologically fragile region and was the major driver for the founding of the United Nations Convention on Combating Desertification and Drought (Zeng, 2003). From the 19th century, the frequency and duration of drought in the region has increased dramatically. Droughts in the 1910, 1940, 1960s, 1970s and the 1980s have led to famines in the region [11,12].

References

1. KEMPER, E. & WAGNER, T. 2004. GIS and Urban Hydrology: Flood Hazard Mapping with GIS Missouri. USA.: Department of Public Works, Storm Water Services Division. Springfield.
2. TXTDOT 2009. Hydraulic Design Manual Texas, USA: Texas Department of Transportation (TxTDOT).

3. ODURO-AFRIYIE, K. & ADUKPO, D. C. 2006. Spectral Characteristics of the Annual Mean Rainfall Series in Ghana, West Africa. *Journal of Applied Ecology,* 9**,** 83-91.
4. NADMO 2009. Hazard Mapping in Ghana. *In:* AMOAKO, P. Y. O. & AMPOFO, S. T. (eds.). Accra, Ghana: National Disaster Management Organization.
5. ASARE-KYEI, D., FORKUOR, G. & VENUS, V. 2015b. Modeling Flood Hazard Zones at the Sub-District Level with the Rational Model Integrated with GIS and Remote Sensing Approaches. *Water,* 7**,** 3531-3564.
6. BBC. 2007. *News Africa* [Online]. Available: http://news.bbc.co.uk/2/hi/6996584.stm [Accessed December 15 2013].
7. WORLD BANK 2009b. Disaster Risk Management Programs for Priority Countries. Washington, D.C 20433, USA: Global Facility for Disaster Reduction and Recovery.
8. USAID 2011. Ghana Climate Change Vulnerability and Adaptation Assessment. *In:* STANTURF, J. A., WARREN, M. L. J., CHARNLEY, S., POLASKY, S. C., GOODRICK, S. L., ARMAH, F. & NYARKO, Y. A. (eds.). United States Agency for Internatioal Development, USDA Forest Service, International Programs.
9. IRIN NEWS. 2009a. *Burkina Faso-Ghana: One country's dam, another's flood* [Online]. Available: http://www.irinnews.org/report.aspx?reportid=86015 [Accessed January 18 2013].
10. IRIN NEWS. 2009b. *Burkina Faso, Hospital patients evacuated post-flooding.* [Online]. Ouagadougou. Available: http://www.irinnews.org/Report.aspx?ReportId=85976 [Accessed January 18 2013].
11. ZENG, N. 2003. Drought in the Sahel. *Science,* 302**,** 999-1000.
12. MISHRA, K. A. & SINGH, V. P. 2010. A review of drought concepts. *Journal of Hydrology,* 391**,** 202-216.
