# Supplementary material for: Development and validation of risk profiles of West African rural communities facing multiple natural hazards
Source: PLoS One. 2017 Mar 1;12(3):e0171921. doi: 10.1371/journal.pone.0171921 (PMC5382969; doi:10.1371/journal.pone.0171921)
Supplement: S2 File — (PDF) [file pone.0171921.s002.pdf]

S2 Text. Exploratory data analysis and bivariate correlation analysis

In the Vea study area, two indicators in the adaptive capacity component were removed from subsequent analysis after the statistical descriptive procedure. The indicator, 'access to national emergency funds' was removed for lack of data whilst the indicator 'local emergency funds as percentage of national budget' was removed due to lack of variability within the community clusters. Similarly, in the Dano study area, two indicators in the adaptive capacity component were removed from further analysis. The indicators, 'social capital' and 'early warning system' were removed for lack of variability within the community clusters. In the Dassari study area, four indicators were removed from further analysis. Two of the indicators, 'prevalence of stunted children under age five' and 'prevalence of wasted children under age five' which belong to susceptibility of the social system were removed for lack of community level data. Furthermore, one coping capacity indicator, 'local emergency funds as a percentage of local budget' and as well as one adaptive capacity indicator, 'Farm labor availability' were removed due to lack of variability within the datasets of the various community clusters.

S.2.1. Bivariate Correlation matrix

Following the approach of Backhaus *et al*. [1] and Damm [2], a bivariate correlation matrix was constructed to understand the strength and direction of the linear relationships between the indicators especially between those indicators in the same component of the framework. The Pearson correlation coefficient was estimated for indicators with absolute metric variables whilst the Spearman correlation coefficient was estimated for indicators with ordinal variables. Similar to the approach of Damm (2010), a rule of thumb was used where all relationships with a coefficient above a threshold value of r=0.65 were carefully scrutinized. The results of this bivariate correlation is presented below. The final indicator set for the Vea area is presented in the main manuscript and those for Dano and Dassari are presented in S1 Fig. and S2 Fig.

In the Vea study area, this approach resulted in the following correlation relationships:

i. The indicator 'Physical infrastructure' is significantly correlated with the indicator 'Insecure settlement' with r=0.9. Since both indicators belong to the same vulnerability sub-component "exposure of social system", one of them is dropped. Physical infrastructure was dropped because the only data available to describe physical infrastructure within the community clusters was road network which could grossly underestimate the number and types of other infrastructure in the communities such as schools and markets.
ii. The indicators 'Protected area' and 'Agricultural area' were significantly correlated (r=0.95). These two indicators belong to the same sub-component "exposure of ecological subsystem" and hence one is redundant and was removed. Protected Area was removed because its retrieval involved considerable uncertainty and thus could not meet the criteria of good data quality.

iii. ‘Unimproved drinking water source’ has a significant correlation with two other indicators belonging to the same component. It correlates significantly with ‘Number of dependents per household’(r=0.68) and ‘Distance to drinking water’ (r=0.78). These double correlations mean that removing ‘Unimproved water source’ will help avoid redundancy.

iv. Again, the indicator ‘Prevalence of poverty’ correlates strongly with ‘Prevalence of stunted children’ (r=0.9). These two indicators belong to the same vulnerability component, yet within this component, they fulfill different analytical purposes and also describe different factors that determines the extent to which a household or community is vulnerable to droughts and floods. Whilst ‘Prevalence of poverty’ belongs to ‘Economic and dependencies’ category of the social system, ‘Children under age five who are stunted’ is a health and nutrition factor. Since there are just two health and nutrition related indicators in the framework, the two indicators were kept. However, Prevalence of stunted children was weighted lower due to its inherent data quality.

In the Dano study area, the following correlation matrixes were observed:

v. The indicator ‘Prevalence of poverty’ exhibits significant correlation with two indicators. It has positive association with ‘Caloric intake per capita’ (r=0.77) and ‘Population density’ (r=0.73). Due to these double correlations exhibited by ‘Prevalence of poverty’, it was removed to avoid redundancy and doubling effects.

In the Dassari study area of Benin, observed relationships are outlined below:

vi. ‘Total soil nitrogen’ correlates with ‘Soil organic matter’ (r=0.68). Since both indicators belong to the capacity sub-component’ Ecosystem robustness’, one of them is redundant and must be removed. ‘Total soil nitrogen’ was removed because of poor data quality.

vii. Again in this study area, ‘Green vegetation cover’ also has a strong correlation with ‘Soil organic matter’ with a coefficient of r = 0.84. Both indicators belong to the component ‘Ecosystem robustness’ and were all retrieved from remote sensing procedures. However, in terms of understandability of the two indicators among practitioners, ‘Green vegetation cover’ was found difficult to be understood and was thus removed from subsequent analysis.

viii. Expectedly, ‘Water holding capacity’ and ‘Infiltration rates’ have a perfect positive relationship (r=1). Since both belong to the same component, ‘Infiltration rates’ which exhibited a lower variability within all community clusters were removed.

ix. Finally, in this study area, ‘Access to agricultural extension service’ and ‘Farm labour availability have a significant negative correlation of r =-0.77. Both of them belong to the adaptive capacity component and subsequently, ‘Farm labour availability’ was removed due to its low rank within the sub-component.

It must be noted that varying degrees of significant correlations were found among other indicators in all the three study areas. However, since they belong to different components of the vulnerability, they are deemed to represent different causes and aspects of vulnerably and thus those relationships were neglected.

Equations used in the methods section.

Equation 1: Normalizing of the indicators

$$I_q = \frac{X_q - Min(X_q)}{Max(X_q) - Min(X_q)}$$

Where I is the normalized indicator and q is each value/observation of the indicator.

Equation 2:Al-Essa model for Converting ranks to weights

$$Wr = 100 - Sn(r-1)$$

Where w_r is the weight of the indicator, r is the rank, and Sn is the absolute value of the slope estimated by least squares regression when the number of indicators is equal to n. Using least-squares regressions to Sn versus n, Al-Essa [3] obtained equation 5 which converts all the ranks provided by the experts into weights.

Equation 3: model for converting rank to weights

$$Wrn = 100 - \left(3.19514 + \frac{37.75756}{n}\right) \times (r-1)$$

Where $1 \leq r \leq n$ and r and n are integer

References

1. BACKHAUS, K., ERICHSON, B., PLINKE, W. & WEIBER, R. 2006. *Multivariate Analyse methoden. Eine anwendungsorientierte Einführung* Berlin, Heidelberg, Springer.
2. DAMM, M. 2010. *Mapping Social-Ecological Vulnerability to Flooding. A sub-national approach for Germany.* PhD, Rheinischen Friedrich-Wilhelms University of Bonn.
3. AL-ESSA, A. 2011. *Evaluating the effectiveness of methods for converting ranking into weights with application for maintenance contractor selection.* ProQuest Dissertations and Theses, King Fahd University of Petroleum and Minerals.
