# Supplementary material for: Development and validation of risk profiles of West African rural communities facing multiple natural hazards
Source: PLoS One. 2017 Mar 1;12(3):e0171921. doi: 10.1371/journal.pone.0171921 (PMC5382969; doi:10.1371/journal.pone.0171921)
Supplement: S3 File — (PDF) [file pone.0171921.s003.pdf]

S3 Text. Results of the main components of vulnerability and risk.

S3.1.Exposure of the rural communities to the multiple hazards

Exposure to hazards is an important dimension of the overall risks faced by a system or community. The implementation of an SES approach means that the exposure index represents both the exposure of the environmental sub-system to droughts and floods as well as the exposure of the social sub-system. In Table A below, the exposure of all the community clusters studied in the three countries have been presented. In the Vea study area (Ghana), the Kula river community cluster is the most exposed community, followed by communities in the Vea main drain and Valley zone in that order. Communities in the Kanga has the least exposure with an index value of just 0.134. Similarly, in the Dano study area (Burkina Faso), communities in the Loffing-Yabogane cluster are the most exposed to the multiple hazards followed by those in Batiara, Bolembar and Gnipkiere in that order. In this study area, Meba Pari has the lowest exposure index of 0.225. Also in the Dassari study area (Benin), Porga cluster of communities are the most exposed followed by Tankouri and Sechendiga clusters.

Table A. Community ranking of the exposure of SES to droughts and floods

	Vea study area		Dano study area		Dassari study area	
**Rank**	**Community cluster**	**Exposure index**	**Community cluster**	**Exposure index**	**Community cluster**	**Exposure**
**1**	Kula river drain	0.581	Loffing-Yabogane	0.591	Porga	0.405
**2**	Vea main drain	0.496	Batiara	0.585	Tankouri	0.269
**3**	Valley zone	0.349	Bolembar	0.554	Setchendiga	0.234
**4**	Balungu	0.341	Gnikpiere	0.551	Nagassega	0.224
**5**	Kolgo-Anateem	0.313	Yo	0.542	Ouriyori	0.222
**6**	Anafobiisi	0.299	Complan	0.535	Firihoun	0.192
**7**	Apatanga	0.297	Tambalan	0.523	Pouri	0.154
**8**	Samboligo	0.297	Dano sector 1,2,4	0.482	Tetonga	0.139
**9**	Soe	0.295	Kpeleganie	0.462	Tigniga	0.121
**10**	Tarongo	0.195	Lare	0.283	Tihoun	0.120
**11**	Beo Adaboya	0.193	Sarba	0.275	Dassari	0.113
**12**	Bongo zone	0.164	Dano sector 7	0.236	Koulou	0.044
**13**	Kanga	0.134	Meba Pari	0.225		

The results show the mean exposure index is highest for communities in the Dano study area (0.45), that for Vea is 0.30 and Dassari communities has mean exposure index of 0.19. Exposure of communities in Dano is also more variable within communities. The variability is estimated at 0.14

around the mean in Dano and 0.12 in Vea. The higher variability of the exposure index in Dano means significant differences exist between the communities in terms of exposure.

It is interesting to note that exposure of communities followed the same pattern of the Flood Hazard Index maps developed by Asare-Kyei *et al.* (2015b) where the distribution of flood hazard in the study areas was modelled. In their study, the Kula River and Vea main drain in Vea; Porga in Dassari and Loffing-Yabogone in Dano were reported to be falling in high flood intensity zones. This study reinforces this finding and shows that the exposure index followed the pattern of flood hazard intensity zones. Although, there are other determinants of exposure as can be seen in the indicators used to construct the index, this fact shows the strong effect proximity to high flood intensity zones has on the overall SES exposure to the hazards. Another major driving factor influencing community exposure to multiple occurrences of drought and flood is the indicator measuring the share of the population engaged in agriculture. This indicator measures populations whose livelihood depends solely on agriculture and which have no other income or food sources. As expected, 72% of people in the Dano area belong to this category of 'Agricultural Dependent Population' (ADP) , 42% in Dassari and Vea having the least number of people (35%) engaged in only agriculture. Although this indicator was ranked second in Dano and first in both Vea and Dassari (Asare-Kyei *et al*., 2015a), its effect on exposure is still significant.

S.3.2. Susceptibility of the communities to drought and floods

Susceptibility is measured as inherent conditions within the communities that predispose them to be adversely affected by the two hazards. The SES approach measures susceptibility for both the socio-economic and environmental sub-systems. Within the social-economic sub-system, four dimensions comprising 'poverty and dependencies', housing, public infrastructure and health and nutrition are considered. Table B below provides details about the susceptibility indices of the communities.

In Table B, the three most susceptible community clusters have been highlighted in grey. Interestingly, all the highly susceptible communities in the Vea area are in the Bongo district. In this study area, Tarongo has the highest susceptibility of 0.693 and Kolgo-Anateem clusters having the least susceptibility. Susceptibility indices in the Dassari area are generally high with a mean of 0.44. Communities in Dano area has a mean of 0.37. However, there are sharp differences in susceptibility indices in the Vea area measured by the standard deviation of 0.12 while communities in the Dassari area record less variability (0.6) from each other.

Table B. Community rankings in terms of susceptibility to the multiple hazards

	Vea study area		Dano study area		Dassari study area	
**Rank**	**Community cluster**	**Susceptibility index**	**Community cluster**	**Susceptibility index**	**Community cluster**	**Susceptibility index**
**1**	Tarongo	0.693	Bolembar	0.534	Setcheniga	0.537
**2**	Samboligo	0.594	Yo	0.506	Tetonga	0.505
**3**	Balungu	0.525	Dano sector 7	0.398	Dassari	0.497
**4**	Bongo zone	0.473	Complan	0.395	Porga	0.494
**5**	Kula river drain	0.468	Loffing-Yabogane	0.379	Tigniga	0.476
**6**	Apatanga	0.438	Dano sector 1,2,4	0.375	Koulou	0.466
**7**	Beo Adaboya	0.406	Gnikpiere	0.368	Firihoun	0.446
**8**	Kanga	0.384	Lare	0.349	Tihoun	0.436
**9**	Anafobiisi	0.382	Sarba	0.334	Tankouri	0.404
**10**	Vea main drain	0.382	Batiara	0.318	Ouriyori	0.398
**11**	Valley zone	0.375	Meba Pari	0.302	Nagassega	0.383
**12**	Soe	0.345	Tambalan	0.290	Pouri	0.343
**13**	Kolgo-Anateem	0.219	Kpeleganie	0.234		
	Mean	*0.437*		*0.367*		*0.448*
	Standard deviation	*0.119*		*0.814*		*0.575*

S3.3. Lack of capacity index

Community lack of capacity to cope and adapt to the hazards occurrence is an integral part of the overall vulnerability of the community. Total lack of capacity in this study has been computed from three sub-indices, lack of coping capacity, lack of ecosystem vitality and lack of adaptive capacity to respond to long-term hazards.

Table C presents the lack of capacities existing within the three study areas. In the Vea area, Samboligo, Apatanga and Soe, all in the Bongo district are the three clusters with the least capacity to cope, adapt and have poor state of the environment. In Dano, Loffing-Yabogane, Yo and Complan are the top three communities with least capacity whilst Tankouri, Firihuou and Tetonga in Dassari area have the least capacity. In terms of capacity, there is no significant difference between the three study areas with mean lack of capacity. All are greater than 50% with minimal differences in variability. Lacks of coping and adaptive capacities are major contributors to the total lack of capacity.

Table C. Community rankings in terms of lack of capacity to cope, adapt and ecosystem vitality

	Vea study area		Dano study area		Dassari study area	
**Rank**	**Community cluster**	**Lack of capacity**	**Community cluster**	**Lack of capacity**	**Community cluster**	**Lack of capacity**
**1**	Samboligo	0.614	Loffing-Yabogane	0.600	Tankouri	0.616
**2**	Apatanga	0.613	Yo	0.586	Firihoun	0.658
**3**	Soe	0.606	Complan	0.582	Tetonga	0.595
**4**	Kolgo-Anateem	0.580	Tambalan	0.551	Ouriyori	0.587
**5**	Balungu	0.544	Batiara	0.524	Pouri	0.564
**6**	Bongo zone	0.534	Kpeleganie	0.499	Tihoun	0.497
**7**	Beo Adaboya	0.532	Sarba	0.495	Porga	0.495
**8**	Vea main drain	0.493	Gnikpiere	0.494	Tigniga	0.481
**9**	Anafobiisi	0.475	Lare	0.485	Nagassega	0.475
**10**	Valley zone	0.465	Bolembar	0.482	Koulou	0.449
**11**	Kanga	0.465	Dano sector 1,2,4	0.478	Dassari	0.438
**12**	Tarongo	0.422	Dano sector 7	0.432	Setcheniga	0.423
**13**	Kula river drain	0.399	Meba Pari	0.374		
	Mean	*0.519*		*0.506*		*0.519*
	Standard deviation	*0. 722*		*0.634*		*0.710*
