# Supplementary figures and images for: Development and validation of risk profiles of West African rural communities facing multiple natural hazards
Source: PLoS One. 2017 Mar 1;12(3):e0171921. doi: 10.1371/journal.pone.0171921 (PMC5382969; doi:10.1371/journal.pone.0171921)

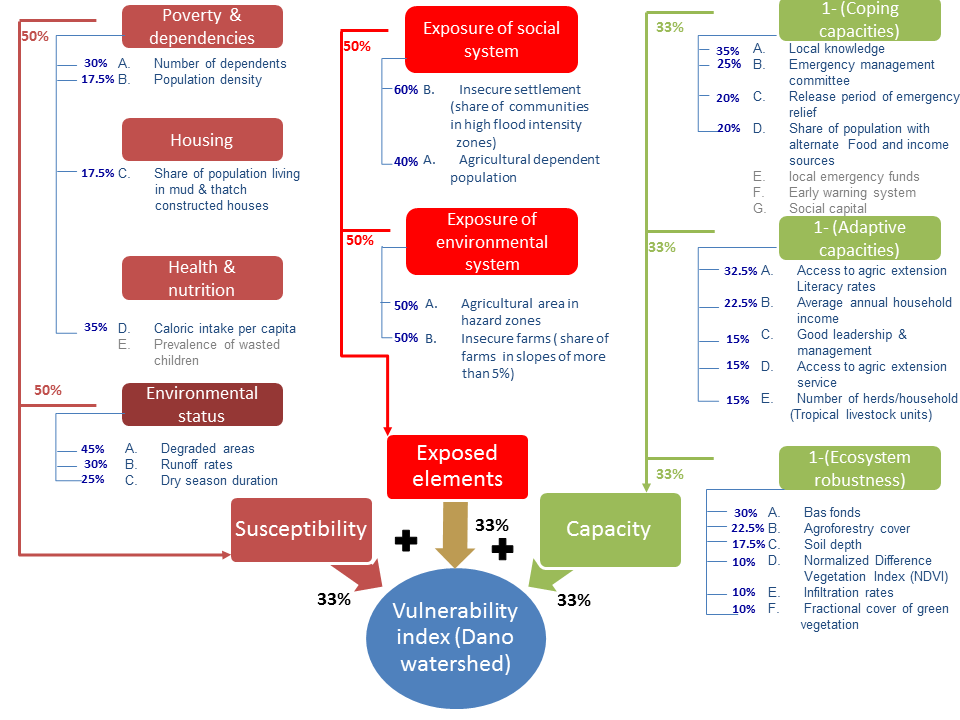

Supplement: S1 Fig — (TIF) [file pone.0171921.s004.tif]

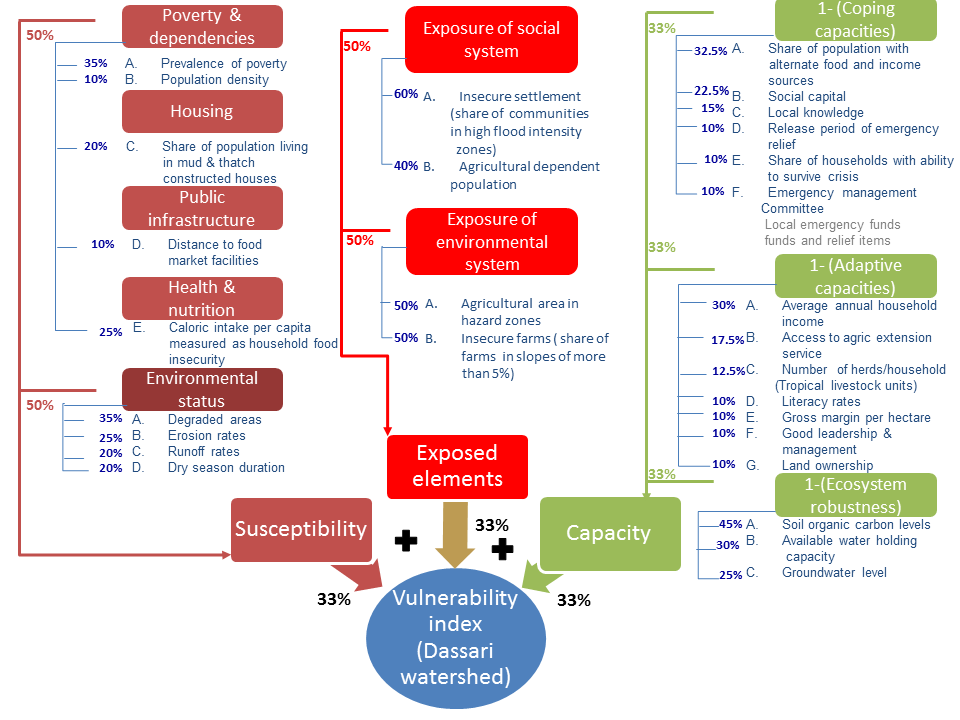

Supplement: S2 Fig — (TIF) [file pone.0171921.s005.tif]
