# Supplementary material for: Development and validation of risk profiles of West African rural communities facing multiple natural hazards
Source: PLoS One. 2017 Mar 1;12(3):e0171921. doi: 10.1371/journal.pone.0171921 (PMC5382969; doi:10.1371/journal.pone.0171921)
Supplement: S1 Table — (PDF) [file pone.0171921.s006.pdf]

S1 Table Construction of indicator data values and data sources

**Vulnerability Component: Exposure**			
**Indicator: rank & applicable study area**	**Definition and Measuring unit**	**Indicator construction and limitation of indicator**	**Data source**
Agricultural dependent population<br>Vea 1/3[1]; Dano 2/2 and Dassari 1/2	The percentage of the area's total population depending on only agriculture related employment (including hunting, fishing and forestry). The number of people with only agriculture as their source of livelihood was divided by the total number of sampled households and scaled from 0 to 1	The survey instrument sought to know if the respondents are engaged in only agricultural activities and has no other source of livelihood. This indicator is valid as several experts believe it gives a better description of people depending on agriculture (Adger 2004).	Own household survey
Insecure settlement:<br>Vea 3/3; Dano 1/2 and Dassari 2/2	Percentage of communities within the cluster which are located in high hazard intensity zones.	Using the flood hazard intensity map developed by Asare-Kyei *et al.* (2015b), in GIS environment, the very high and high intensity flood zones were considered. The process begun by intersecting the three vector layers, the flood index map, land cover and slope to determine land cover types under two intensity zones. After intersecting, a new field is added and area in hectares was calculated. The community cluster maps were used to clip the intersected features to allow for community level analysis. Then the total area occupied by each community cluster was estimated using the summarize tool in ArcGIS. Then, the flood intensity zone field was sorted in descending order and the very high and high zones were selected. The summarized tool was again used to calculate	Asare-Kyei *et al. (2015b)*

[1] These numbers represent the rank of the indicator within the vulnerability sub-component. In this case, Agricultural dependent population is ranked as the first out of three indicators in the Vea study area.

		the area of the respective land covers that fall in the two hazard intensity zones.	
Physical infrastructure<br>Vea 2/3	Number of physical infrastructure in an area. Such as irrigation dams, hospitals, schools, food markets and major bridges located in floodplains	Physical infrastructure was estimated using road network map of Ghana. Each community cluster was used to extract the very high and high areas of the flood intensity map and then also the road network map. The clipped flood intensity map and road network map were intersected in GIS to determine the percentage of the road network in a community cluster that falls within the two high flood intensity zones.<br>Lack of local level data means only road network could be used to describe the physical infrastructure located in flood plains.	Asare-Kyei *et al.(2015b) and Road network map from Ghana basemaps*
Insecure Farms<br>Vea 3/3; Dano 2/2	Percentage of cropland within the community cluster located in slopes of more than 5%.	Retrieval of data values for these indicators follow the approach used to construct the data values for the indicator "Insecure settlement" describe above.	From 30m spatial resolution Global Digital Elevation Model developed jointly by the Japanese Ministry of Economy, Trade and Industry (METI) and the United States National Aeronautics and Space Administration (NASA).
Agricultural Area<br>Vea 1/3; Dano 1/2; Dassari 2/2	Percent of total land used for agricultural activities in an area located in flood plain. This includes arable land and pastures in flood plains		LULC maps for the three study areas were generated by classifying high spatial resolution (5m) multi-temporal RapidEye images developed by (Forkuor et al., 2014). Flood map from Asare-Kyei *et al*. (2015b).
Protected Area<br>Vea 2/3; Dassari 1/2	Percent of area of land that are protected including national parks, forest reserves, watersheds etc located in flood plains		Same as above

Vulnerability Component: Susceptibility of social system			
**Indicator:**	**Definition and Measuring unit**	**Indicator construction and Validity/limitation of indicator**	**Data Source**
Number of dependents: Vea 1/10, Dano, 4/7	Average number of household members below the age 15 and above the age of 65.	This is retrieved from household survey data where the number of household members below the age of 15 and above 65 years were added and divided by the total number of households sampled in a community cluster. High number of dependents population per household denotes high vulnerability as such individuals rely on family members or social services for financial services and other support.	From own household survey
Population density: Vea 2/10, Dano 7/7, Dassari 8/8	This is the number of people per square kilometer in the inhabited area of the study areas. In Dano and Dassari study areas, the original indicator, Demographic pressure was replaced with the population density.<br>High population density increases vulnerability.	Population density data at 100m resolution estimated in 2013 for the year 2015 was retrieved. The data has been adjusted for UN national population estimates. This data was extracted as ESRI shapefile and overlaid on the community cluster maps. Geoprocessing techniques were used to estimate average population density per community cluster.	Secondary data from Africa Population database (AFRIPOP) was used. Details about can be found at: http://www.worldpop.org.uk/data/summary/?contselect=Africa&countselect=Ghana&typeselect=Population
Quality of Housing: Vea 3/10, Dano 5/7, Dassari 5/8	Percentage of households within a cluster living in houses prone to flood damage and or bushfires. Higher percentage increases vulnerability.	This is also termed percent of poor housing. Poor housing includes mud and thatch with no concrete and proper roofing system. The percentage of people living in mud and thatch house or mud with aluminum roofing sheets were computed.	From own household survey data
Distance to water: Vea 4/10	Percentage of total households within a community cluster that travel more than 30 minutes for drinking water. Higher percentage increases vulnerability.	Respondents were asked about the time spent in getting to the nearest water source.	From own household survey data

Distance to food market: Vea 5/10, Dassari 7/8	Percentage of households within a community cluster that travels for more than 30 minutes to reach the nearest food market. High percentage increases vulnerability.	Respondents were asked about the time spent in getting to the nearest food market to either sell farm produce or buy foodstuffs.	From own household survey data
Prevalence of stunted children: Vea 7/10	Percent of children under 5 in a community cluster who are stunted (have low height for their ages). Higher percentage denotes higher vulnerability.	The USAID METSS project conducted a Population Based Survey (PBS) of key socio-economic variables. The data is available at the district scale and was thus downscaled to the community clusters. Prevalence of poverty was assumed to directly affect stunting and therefore poverty scores in the clusters were used as weighting factors to derive stunting values from the district stunting data.	From secondary data which has been collected by United States Agency for International Development (USAID) funded METSS project in Ghana
Caloric intake per Capita: Vea 8/10, Dano 2/7, Dassari 4/8	The dietary energy consumption per person is the amount of food, in kcal per day, for each individual in the total population. The study couldn't directly measure this indicator in the field and so Household food insecurity was used as a proxy. High percentage denotes higher vulnerability	Following the approach of World Food Program (WFP, 2012), household food insecurity is measured as a percentage of households classified as severely food insecure and moderately food insecure. Using non-food income, total crop production from all crops produced by the household and Tropical Livestock Unit (TLU), each of these variables were ranked and divided into quintiles (5 equal parts). The scores were subsequently multiplied and the final total score divided into 4 parts. This means the households have been classified into 4 food security levels. Households with severe and moderate food insecurity was computed for each cluster (WFP, 2012).	Data source for estimating household food insecurity is from own household survey
Female headed households: Vea 10/10	Percentage of total households in a community cluster that is headed by a female. High percentage denotes high vulnerability.	Respondents were simply asked to indicate the head of the household by sex.	From own Household survey data

Prevalence of poverty: Vea 9/10, Dassari 1/8	Percentage of households living below the national absolute poverty line. High percentage increases vulnerability.	Household equivalent scale was used as weighting factor for household size. Then all income sources including non-farm income and farm income were added. Absolute national poverty line estimated by Ghana Statistical Service (GSS) in 2014 as Ghc3.6/person per day was used for the Vea study and national absolute poverty line in Benin estimated in 2003 ass FCFA82, 672 was used for the Dassari study area. The percentages of poverty levels in Dassari are relatively low probably because the national poverty line is outdated.	Household survey data
Household size: Dano 3/7	Average number of total household members in a community cluster	From household survey data. Respondents were asked to indicate the total number of people in the household.	From own Household survey data

Vulnerability Component: Susceptibility of ecological sub-system			
**Indicator:**	**Definition and Measuring unit**	**Indicator construction and limitation of indicator**	**Data sources**
Degraded areas: Vea 1/2, Dano 1/3, Dassari 1/4	Percentage of land in the community cluster that is degraded or deserted.	The land degradation classes 'map shows the complete status in provision of biophysical ecosystem services and the processes of declining biophysical ecosystem services by considering the combined value of each biophysical axis' (FAO LADA). The land degradation dataset in Geotiff format was imported into ArcGIS for analysis. Of the eight classes listed in the GLADIS database, five were used to compute the percent degraded area per community cluster. These classes are:<br>a) low status, medium to strong<br>b) high status, medium to strong<br>c) low status, weak degradation<br>d) low status improving and<br>e) barelands.<br>A key limitation of the datasets is its spatial resolution. At a spatial resolution of 9km, the dataset is not ideal for local scale assessment but no better dataset could be found.	Data was obtained from FAO LADA project hosted at the Global Land Degradation Information System (GLADIS) database. For details see LADA (2011), http://www.fao.org/nr/lada/gladis/glad_ind/
Runoff rates: Dano 2/3, Dassari 3/4	Surface runoff measured in mm/hour is the flow of water that occurs when the soil is infiltrated to full capacity and excess water from rain flows over the land. Higher runoff increases vulnerability of ecological system.	Runoff was estimated by applying the rational model integrated with remote sensing and GIS techniques	Data source from Asare-Kyei *et al*. (2015b).

Crop type:<br>Vea 2/2	This indicator was originally defined in Asare-Kyei et al (2015a) as percent of community cluster under cultivation of drought and flood sensitive crops. However, this was difficult to operationalize and hence the variable "lack of crop diversification" was used as a proxy. Higher percentage increases vulnerability.	Lack of crop diversification measures the percentage of households in a community cluster having three or less different crops under cultivation in any farming season. This was estimated by counting the number of different farm plots of different crops cultivated by sampled farmers and deriving the average per cluster. Relationship between crop diversification and adaptive capacity/vulnerability can be found in (Tarleton, M., & Ramsey, D. 2008; Ngigi 2009).	From own household survey
Dry season duration:<br>Dano 3/3,<br>Dassari 4/4	The average duration in days of the dry season over the last decade. This was operationalized by using the frequency of irregular rainfall events. Higher occurrence or irregular rainfall events increases vulnerability.	This was operationalize with the frequency of irregular rainfall recorded over the period, obtained from household surveys. Responses were converted to categorical variable as follows:<br>a) 6 represents irregular rainfall event every year<br>b) 5 represents irregular rainfall occurrence once every two years<br>c) 4 is once in three years irregular rainfall<br>d) 3 is once in four years irregular rainfall<br>e) 2 is once in five years and<br>f) 1 represents once in more than 5 years.<br>This sort of measures of the return period of drought events -	From own household survey
Erosion rates:<br>Dassari 2/4	Amount of water erosion recorded in each community cluster measured in tons/ha/year. High erosion rates increases vulnerability	This dataset was retrieved from FAO LADA project database (GLADIS) as described above.	Data was obtained from FAO LADA project hosted at the Global Land Degradation Information System (GLADIS) database. For details see LADA (2011), http://www.fao.org/nr/lada/gladis/glad_ind/

Vulnerability Component: Capacity, ecosystem robustness			
**Indicator**:	**Definition and Measuring unit**	**Indicator construction and /limitation of indicator**	**Data source**
Soil Organic Matter (SOM): Vea 1/5, Dano 4/8, Dassari 1/6	The amount of Soil Organic Carbon held per unit area of land per year. Soil organic carbon content (fine earth fraction) in 2.5cm (mean estimate) depth (topsoil) was used.<br>Higher SOM levels decreases vulnerability.	SoilGrids1km provides a collection of updatable soil property and class maps of the world at a relatively coarse resolution of 1 km. This data is derived from state-of-the-art model-based on statistical techniques including "3D regression with splines for continuous soil properties and multinomial logistic regression for soil classes". In this study, the SOM was sub-setted and extracted into GIS and the areas of the various community clusters were intersected to determine the average amount of SOM per square km in each cluster. This dataset has a limitation of limited spatial accuracy and contain artefacts and missing pixels. However, they presented the best options of readily accessible data in this category in the study areas. For details see ISRIC (2013).	This data was obtained from SoilGrids1km which is a global soil data product generated at ISRIC - World Soil Information (http://soilgrids1km.isric.org).
Water holding capacity: Vea 4/5, Dano 7/8, Dassari 3/6	This is the amount of 'Water in Millimeters stored in or at the land surface and available for evapotranspiration' (IPCC, 2012). High water capacity reduces vulnerability.	Available water capacity from regridded HWSD is used here. Categorical values use are indicated below:<br>a) 7 = 150mm<br>b) 6 = 125mm<br>c) 5 = 100mm<br>d) 4 = 75mm<br>e) 3 = 50mm<br>f) 2 = 15mm<br>g) 1 = 0mm	Data taken from rigridded Harmonized World Soil Database (HWSD) (FAO, 2009)
Bas Fonds: Dano 1/8	The number of reservoirs and water bodies (bas-fonds) located in the study area. Operational definition adopted here is the percentage of the cluster's total area suitable for bas-fonds management. Higher percentage reduces vulnerability.	This is derived from International Water Management Institute (IWMI) bas fonds management suitability maps, Category one on the map representing areas highly suitable for bas fonds management was extracted and used. This is expressed as a percentage of the total land area within the cluster that are described as highly suitable for bas fonds management.	Details about this is found at FAO (2012).

Infiltration rate: Vea 2/5, Dano 6/8	The rate measured in Millimeters per hour at which soil absorbs rainfall or irrigation water. This indicator could not be measured in the field due to time constrains and a proxy, Drainage class was used. High drainage class values denote reduced vulnerability.	The study used Drainage class as proxy. This is a 1km resolution soil map from the Harmonized World Soil Database (HWSD) version 1.1 produced in 2009 by the International Institute for Applied System Analysis (IIASA). The HWSD is an image file linked to a comprehensive attribute database in Microsoft Access. This attribute information includes soil mapping units, soil texture for top and sub soils and several other soil properties including Drainage. There are 7 drainage classes in this database. In this study, the 7 classes were converted to categorical values as follows:<br>a) very poor, excessive = 1<br>b) poor = 2<br>c) Imperfectly, somewhat excessive = 3<br>d) moderately well = 4<br>e) well = 5<br>Details about this database can be found in FAO (2009).	This is a 1km resolution soil map from the Harmonized World Soil Database (HWSD) version 1.1 produced in 2009 by the International Institute for Applied System Analysis (IIASA).
Green Vegetation Cover (GVC): Vea 5/5, Dano 8/8	Fractional cover of green vegetation during the dry season. Higher GVC reduces vulnerability.	Green Vegetation was computed from 1 km MODIS-based Maximum Green Vegetation Fraction. These data describe annual maximum green vegetation fraction (MGVF), and are based on 12 years (2001-2012) of Collections of 5 MOD13A2 Normalized Difference Vegetation Index (NDVI) data. The data is based on the annual maximum NDVI and linear mixing models that describe green vegetation fraction (vs. non vegetated area) for each land cover class for each year. Generation of these data is described in Broxton et al., 2014b. The data has been re-gridded from the MODIS sinusoidal grid to a regular latitude-longitude grid. Details at: Broxton *et al*. (2014). Average GVC for each community cluster was computed with geostatistical techniques in GIS.	Details at: Broxton *et al*. (2014).

Groundwater level (GWL): Vea 3/5, Dassari 6/6	Average level at which most boreholes in the area reaches water. This is measured in meters below ground level. Lower GWL denotes reduced vulnerability	The WRI conducted Hydro-geological Assessment Project to monitor the water levels of 37 observation boreholes throughout the three northern regions since 2005. Using the mean water level in cm recorded between 2005 and 2011, the 37 observation points were interpolated with Kriging method in GIS to obtain data for all community clusters. To follow the general trend of data in this vulnerability sub-component, the GWL data have to be ranked. Ranking was done by sorting the GWL data in descending order. The area with the highest GWL was given a lowest value of 1 and the area with the lowest GWL was given a highest value of 13. This is based on theoretical understanding that areas with lower groundwater levels offer more water access to communities in times of climate change and these will have more capacity to cope or adapt (less energy required to extract water, less costs to dig wells).<br>Data values are categorical values representing meters below ground level (mbgl) as follows:<br>a) 1 = >250<br>b) 2= 100 to 250<br>c) 3 =50 to 199<br>d) 4 = 25 to 50<br>e) 5 = 7 to 25 and<br>f) 6 = 0 to 7.<br>The higher the categorical score the better in terms of access to groundwater and thus increases community capacity to cope with limited access in the face of climate change. Thus a community with a score of 6, means depth to groundwater is relatively shallow, depth range 0 to 7 mbgl and will normally has access to more water in the event of drought.	In the Vea study area of Ghana, GWL data was obtained from the Water Research Institute (WRI) of Ghana.<br>In the Dassari study area, the GWL data was obtained from the British Geological Survey of Africa wide groundwater mapping project (Macdonald et al., 2012).

Agroforestry cover: Dano 2/8	The percentage of total land in the community cluster under agroforestry plantation or of considerable tree density.	Where respondents were asked to indicate if they practice agroforestry system. Farming practices where 10 or more/acre economic trees such as Shea and Baobab are purposely left in the farms were also counted as agroforestry system.	From own household survey
Soil depth: Dano 3/8	The maximum rooting depth at which major crops can grow. This is operationalized as the depth to bedrock in centimeters.	This data is obtained from ISRIC-World Soil Information as described above.	From ISRIC- World Soil Information as described above.
Normalized Difference Vegetation Index (NDVI): Dano 5/8	Normalized difference vegetation index during peak crop growth	This follows the computational description of Green Vegetation Cover described above.	

Vulnerability Component: Capacity, Coping capacity			
**Indicator:**	**Definition and Measuring unit**	**Indicator construction and /limitation of indicator**	**Data source**
Alternate food and income source: Vea 1/7, Dano 7/7, Dassari 1/7	Percentage of population in a community cluster with additional food and income source other than agriculture. Higher percentage increase capacity and reduces vulnerability	This is from household survey data and it's computed as percent of households with alternate food and income sources. Computed by adding percent with alternate income sources and percent with outside family support.	From Household survey
Ability to survive crisis: Vea 2/7, Dassari 6/7	The percentage of total households within a community cluster that are able to survive crisis. Higher percentage reduces vulnerability.	From household survey data. Respondents were asked about their sense of security. Household who feel insecure or somewhat insecure are not able to survive crisis. . Households that feels either "somewhat" or "very" insecure about their ability to withstand any hardships have low coping capacity.	From household survey
Social capital: Vea 3/7, Dassari 2/7	Percentage of communities within a cluster with highly or adequate participation of people in communal activities such as clean-up campaigns, village meetings etc. Higher ordinal score increases coping capacity and reduces vulnerability.	This is from household survey and focus group discussion. Community leaders were asked to rank the level of participation of community members in communal activities. Four ordinal classes were used:<br>a) total apathy of community members =1<br>b) barely adequate participation = 2<br>c) adequate participation =3<br>d) highly participatory =4	From household survey
Local knowledge: Vea 4/7, Dano 3/7, Dassari 3/7	The percentage of people in a community cluster with good knowledge of climate variability, local environmental issues and have taken part in any disaster risk reduction education in the last five years. Higher percentage reduces vulnerability.	From household survey data. Households were asked to indicate their knowledge on local environmental issues, disaster risk reduction, climate change adaptation and awareness of climate variability. Households who described their knowledge level as high and very high were computed as having adequate understanding of local climate change issues.	From own household survey

Emergency management committee (EMC): Vea 5/7, Dano 4/7, Dassari 7/7	Annual meeting frequency of local emergency committees in the community cluster. Higher meeting frequencies reduces vulnerability	From household surveys and focus group discussion. It was difficult for the disaster volunteers to estimate the number of times they meet in a year and therefore an operational definition of the indicator was found. The indicator was operationalized as a binary variable with two indicating the presence of emergency committees and 1 representing absence thereof. In a cluster of communities, the dominant response was used. For instance, in a cluster of 7 communities, if 5 out of the 7 communities indicate they that they have EMC, that average response was used to represent the cluster.	From own household survey
Relief period of emergency items: Dano 6/7, Dassari 4/7	The length of time in days it takes for disaster managers to provide relief items and emergency support services to affected people. Relief items could include medicines, temporal shelters, blankets, food aid etc. in times of emergencies. High categories increases coping capacity and reduces vulnerability.	This indicator from field surveys measures access to national emergency funds and relief items. Relief response is the response time that disaster managers takes to provide relief to affected people. It is stated in days and converted into categorical variables as values:<br>a) 6 = 1 to 7 days after disaster<br>b) 5 =8 to 15 days after disaster<br>c) 4 = 16 to 30 days after disaster<br>d) 3 = 31 to 60 days after disaster<br>e) 2 = 61 to 300 days after disaster<br>f) 1 = beyond 300 days after disaster	From own household survey

Vulnerability Component: Capacity, Adaptive capacity			
**Indicator:**	**Definition and Measuring unit**	**Indicator construction and limitation of indicator**	**Data sources**
Access to agricultural extension service: Vea 1/7, Dano 4/5, Dassari 2/8	Average number of agriculture extension officers per community in the cluster. High number increases adaptive capacity and reduces vulnerability.	From household survey	
Household income per annum: Vea 2/7, Dano 2/5, Dassari 1/8	Average household income per annum in the community cluster. Higher income decreases vulnerability.	From household survey data. All income sources from all farm plots cultivated by the households, income from sales of livestock and poultry, non-farm income from activities of all economically active household members as well as remittances and support received from friends and family were computed.	From own household survey
Literacy rates: Vea 3/7, Dano 1/5, Dassari 4/8	The percentage of the cluster's household heads that can read and write. Higher percentage increases adaptive capacity and decrease vulnerability.	From field surveys: Initially, the illiteracy rates computed from percentage of households heads who can neither read nor write was estimated from people without any education both formal and informal. This was subsequently subtracted from one to give an indication of percent literate.	From own household survey

Number of herds per household: Vea 4/7, Dano 5/5, Dassari 3/8	Average number of herds of livestock owned by households. Herds include goats, sheep, poultry, cattle and donkeys if they are used for economic activities. Higher herds per household increases adaptive capacity and reduces vulnerability.	From household surveys. The number of all livestock and poultry including cattle, sheep, goats, pigs, chicken, guinea fowls, ducks, dogs and donkeys were recounted by households. These absolute numbers were converted to a common scale to allow for comparison using the Tropical Livestock Units indicated below:<br>a) Cattle = 0.8<br>b) Sheep, goats = 0.1<br>c) Pigs = 0.3<br>d) Chicken, guinea fowl, ducks = 0.007<br>e) Donkey = 0.5	From own household survey
Gross margin per hectare: Vea 5/7, Dassari 5/8	This is the ratio of the difference between total crop revenue and variable production cost per hectare. Higher Gross margin increases adaptive capacity and reduces vulnerability.	From household surveys. Production information for all crops produced by the household was collected. This information included area cultivated per crop, yield/ha, market prices of the commodities and production cost.<br>Gross margin was estimated as total crop revenue less the variable cost of production. Variable cost for gross margin estimation is the sum of all inputs which cost constitutes more than 5% of the total production cost.<br>Sum of gross margins from three most important crops in terms of area under production were then estimated to derive the Gross margin/ha.	From own household survey

Good leadership & management: Vea 6/7, Dano 3/5, Dassari 6/8	Percentage of communities within a cluster with well functional institutional network comprising well respected chiefs and effective local government structures.<br>Higher categorical values increases adaptive capacity and reduces vulnerability.	This is from field surveys. Community members were asked to indicate the level of effectiveness of local government structures and tribal chiefs in managing the affairs of the community especially in times of emergencies. Four ordinal variables were ranked. These are classified as follows:<br>a) 1 is nonfunctional local leadership<br>b) 2 is ineffective local leadership<br>c) 3 is effective local leadership and<br>d) 4 is highly effective local leadership	From own household survey
Access to farm labour: Vea 7/7	Percent of households within a cluster with timely access to labour for major farm activities. Higher percentage increases adaptive capacity and reduces vulnerability	This is from household surveys. Respondents were asked to indicate whether they have immediate access to labour for major farm operations in a situation where funding is not a constraint.	
Access to land or land ownership: Dassari 7/8	Percentage of households within a cluster with unhindered access to land. Higher percentage increases adaptive capacity and reduces vulnerability.	From household surveys. Respondents were asked to indicate whether they own their farmlands or have readily access to farmland to rent especially in settler communities where the people do not own land.	From own household survey
