# Supplementary material for: Development and validation of risk profiles of West African rural communities facing multiple natural hazards
Source: PLoS One. 2017 Mar 1;12(3):e0171921. doi: 10.1371/journal.pone.0171921 (PMC5382969; doi:10.1371/journal.pone.0171921)
Supplement: S2 Table — (PDF) [file pone.0171921.s007.pdf]

S2 Table. Variables used to develop the community impact score

Community cluster	Study area	P-droughts	P-floods	P-multi	Human loss	Housing	Eco-value	cropland	livestock	impact score
**Anafobiisi**	Vea	100.00	92.86	40.00	-	30.00	10,541.00	88.50	323.00	1,536.00
**Apatanga**	Vea	100.00	50.00	42.86	2.00	11.00	8,420.00	78.00	81.00	384.00
**Balungu**	Vea	100.00	20.00	50.00	-	43.00	4,050.00	102.00	47.00	48.00
**Beo Adaboya**	Vea	100.00	93.33	30.00	2.00	24.00	9,430.00	58.00	31.00	128.00
**Bongo zone**	Vea	100.00	82.35	52.94	-	15.00	32,949.00	110.50	159.00	576.00
**Kanga**	Vea	100.00	20.00	20.00	1.00	25.00	15,728.00	51.00	51.00	32.00
**Kolgo-Anateem**	Vea	100.00	6.00	60.00	4.00	80.00	20,110.00	25.00	141.00	648.00
**Kula river drain**	Vea	100.00	100.00	87.10	6.00	120.00	10,499.00	129.75	200.00	24,576.00
**Samboligo**	Vea	93.33	80.00	60.00	4.00	86.00	2,050.00	85.00	12.00	729.00
**Soe**	Vea	100.00	6.67	60.00	-	118.00	25,951.00	58.50	91.00	576.00
**Tarongo**	Vea	92.86	40.00	28.57	-	75.00	7,891.00	51.50	134.00	144.00
**Valley zone**	Vea	84.62	76.92	53.85	-	25.00	11,040.00	57.50	52.00	36.00
**Vea main drain**	Vea	100.00	100.00	80.00	3.00	104.00	9,399.00	85.00	225.00	13,824.00
**Batiara**	Dano	71.43	100.00	57.14	-	17.00	278,571.43	13.00	-	96.00
**Bolembar**	Dano	90.00	100.00	78.00	-	27.00	353,125.00	59.00	34.00	4,608.00
**Complan**	Dano	62.50	75.00	25.00	-	18.00	308,333.33	44.00	35.00	144.00
**Dano sector 1,2,4**	Dano	100.00	83.33	41.67	-	13.00	379,166.92	20.00	4.00	192.00
**Dano sector 7**	Dano	92.86	100.00	33.00	1.00	10.00	83,923.08	16.00	12.00	96.00
**Gnikpiere**	Dano	83.33	100.00	78.00	-	24.00	150,000.00	66.80	37.00	2,304.00
**Kpeleganie**	Dano	90.00	80.00	60.00	-	17.00	150,000.00	16.50	13.00	108.00
**Lare**	Dano	90.00	66.00	40.00	-	3.00	150,000.00	47.50	10.00	36.00
**Meba Pari**	Dano	100.00	60.00	33.00	-	8.00	148,285.71	9.75	12.00	16.00
**Sarba**	Dano	80.00	80.00	80.00	3.00	6.00	172,500.00	9.00	1.00	72.00
**Tambalan**	Dano	100.00	100.00	66.67	-	12.00	185,000.00	16.00	11.00	192.00
**Loffing-Yabogane**	Dano	95.00	80.00	100.00	-	27.00	275,000.00	59.50	31.00	6,144.00
**Yo**	Dano	100.00	100.00	100.00	1.00	22.00	1,115,875.00	35.40	32.00	12,288.00
**N=26**										
