# Supplementary material for: Development and validation of risk profiles of West African rural communities facing multiple natural hazards
Source: PLoS One. 2017 Mar 1;12(3):e0171921. doi: 10.1371/journal.pone.0171921 (PMC5382969; doi:10.1371/journal.pone.0171921)
Supplement: S3 Table — (PDF) [file pone.0171921.s008.pdf]

S3 Table. Indicator reference table for West Africa risk assessment.

Vulnerability sub component	Indicator	Ghana	Burkina Faso	Benin
Exposure of social system	Agricultural dependent population	√	√	√
	Insecure settlement	√	√	√
Exposure of environmental system	Agricultural area in hazard zones	√	√	√
	Insecure farms (cropland in high slopes areas)	√	√	x
	Protected area in hazard zones	x	x	√
Susceptibility of social system	Number of dependents	√	√	x
	Population density	√	√	√
	Quality of housing	√	√	√
	Distance to drinking water source	√	x	x
	Distance to food market	√	x	√
	Prevalence of stunted children	√	x	x
	Caloric intake per capita	√	√	√
	Prevalence of poverty	√	x	√
	Female headed households	√	x	x
Susceptibility of ecological system	Degraded areas	√	√	√
	Crop type (crop diversification practices)	√	x	x
	Runoff	x	√	√
	Dry season duration	x	√	√
	Erosion rates	x	x	√
Capacity – ecosystem robustness	Soil organic matter	√	x	√
	Infiltration rates	√	√	x
	Groundwater level	√	x	√
	Water holding capacity	√	x	√
	Green vegetation cover	√	√	x
	Bas fonds	x	√	x
	Agroforestry cover	x	√	x
	Soil depth (distance to bedrock)	x	√	x
	Normalized Difference Vegetation Index	x	√	x
Coping capacity	Alternative food and income sources	√	√	√
	Ability to survive crisis	√	x	√
	Social capital	√	x	√
	Local knowledge	√	√	√
	Emergency management committee	√	√	√
	Relief period of emergency items	x	√	√
Adaptive capacity	Access to agric and health extension officers	√	√	√
	Average annual household income per capita	√	√	√
	Literacy levels	√	√	√
	Tropical livestock units (Number of herds)	√	√	√
	Gross margin per hectare	√	x	√
	Farm labour availability	√	x	x
	Access to farmland	x	x	√
